# MutSeqR: an open source R package for standardized analysis of error-corrected next-generation sequencing data in genetic toxicology

**DOI:** 10.1093/bioadv/vbaf265

**Published:** 2025-10-23

**Authors:** Annette E Dodge, Andrew Williams, Danielle P M LeBlanc, David M Schuster, Elena Esina, Charles C Valentine, Jesse J Salk, Alex Y Maslov, Chris Bradley, Carole L Yauk, Francesco Marchetti, Matthew J Meier

**Affiliations:** Environmental Health Science and Research Bureau, Health Canada, Ottawa, ON K1A 0K9, Canada; Department of Biology, University of Ottawa, Ottawa, ON K1N 6N5, Canada; Environmental Health Science and Research Bureau, Health Canada, Ottawa, ON K1A 0K9, Canada; Environmental Health Science and Research Bureau, Health Canada, Ottawa, ON K1A 0K9, Canada; Department of Biology, University of Ottawa, Ottawa, ON K1N 6N5, Canada; Department of Biology, University of Ottawa, Ottawa, ON K1N 6N5, Canada; Fulcrum Genomics LLC, Somerville, MA 02144, United States; Division of Hematology and Oncology, University of Washington School of Medicine, Seattle, WA 98195, United States; Department of Genetics, Albert Einstein College of Medicine, Bronx, NY 10461, United States; Matter Bioworks, Brooklyn, NY 11237, United States; MutagenTech Corp, New York, NY 10014, United States; Department of Biology, University of Ottawa, Ottawa, ON K1N 6N5, Canada; Environmental Health Science and Research Bureau, Health Canada, Ottawa, ON K1A 0K9, Canada; Department of Biology, Carleton University, Ottawa, ON K1S 5B6, Canada; Environmental Health Science and Research Bureau, Health Canada, Ottawa, ON K1A 0K9, Canada; Department of Biology, University of Ottawa, Ottawa, ON K1N 6N5, Canada

## Abstract

**Motivation:**

Error-corrected next-generation sequencing (ECS) methods are increasingly used to assess mutagenicity and other genetic toxicology endpoints. The lack of open and standardized bioinformatic workflows and tools poses challenges to data reproducibility, comparability, and consistency in interpretation for its application in genetic toxicity assessment.

**Results:**

We present MutSeqR, an open source R package to analyse ECS mutation data for genetic toxicology studies. MutSeqR offers practical variant filtering, comparative analysis of mutation frequency between experimental conditions, dose–response assessment via benchmark dose calculations, mutation spectrum analysis, and clonality analyses. We demonstrate MutSeqR’s application using published datasets on mice treated with benzo[a]pyrene or benzo[b]fluoranthene, analysed using Duplex Sequencing and SMM-seq, respectively. MutSeqR’s flexible functions enable reproducible analyses across ECS platforms, facilitating research and regulatory applications in mutagenicity testing.

**Availability and implementation:**

MutSeqR is freely available under an open source license at https://github.com/EHSRB-BSRSE-Bioinformatics/MutSeqR. Implemented in R (version 3.4.0 or greater), it supports all major operating systems. Sequencing data for Project 1 has been deposited in the Sequence Read Archive under accession number PRJNA803048. Variant call files for Project 2 are available on Mendeley Data (doi: 10.17632/65dnysxym8.1).

## 1 Introduction

Assessing xenobiotics for mutagenicity is vital to human health, as somatic mutagenesis contributes to many diseases (Jackson *et al.* 2018, Erickson 2003, Godschalk *et al.* 2020), including cancer, and germline mutations can cause hereditary diseases (Beal *et al.* 2017, Marchetti *et al.* 2020, Nguengang Wakap *et al.* 2020). Thus, human and environmental risk assessments require mutagenicity testing of existing and new substances (Krewski *et al.* 2010). Error-corrected next-generation sequencing (ECS) is an emerging, high-throughput method for the direct measurement of mutations in any DNA-based model system (Marchetti *et al.* 2023, Menon and Brash 2023). ECS quantifies spontaneous and genotoxicant-induced mutations and provides in-depth characterization of mutational spectra (Valentine *et al.* 2020, LeBlanc *et al.* 2022, Cho *et al.* 2023, Dodge *et al.* 2023, Schuster *et al.* 2024). ECS enables mechanistic insights and accurate quantification of mutation frequency, and holds potential to advance the translation of mutagenicity testing on xenobiotics (using cell culture and animal models) for human health risk assessment.

ECS improves sequencing accuracy by grouping sequence reads derived from the same DNA molecule to subtract artifacts introduced during library preparation and sequencing (Salk and Kennedy 2020). This enables the detection of ultra-low frequency mutations within large populations of non-mutant DNA molecules. ECS technologies are being developed for a broad range of applications, including cancer biology (Kennedy *et al.* 2019, Valentine *et al.* 2020, Abbasi and Alexandrov 2021, Kostecka *et al.* 2022) and genetic toxicology (LeBlanc *et al.* 2022, Bercu *et al.* 2023, Cho *et al.* 2023, Dodge *et al.* 2023, Smith-Roe *et al.* 2023, Schuster *et al.* 2024). ECS strategies include single-strand (e.g. Safe-SeqS, SiMSen-Seq), tandem-strand (e.g. o2n-Seq, SMM-Seq) and double-strand [e.g. Duplex Sequencing (DS), PECC-Seq, Jade-Seq, PacBio HiFi, CODEC, etc.] consensus sequence methods. Researchers are advocating for the regulatory adoption of ECS for genetic toxicology and cancer risk assessment of xenobiotics (Marchetti *et al.* 2023).

**Figure 1.**
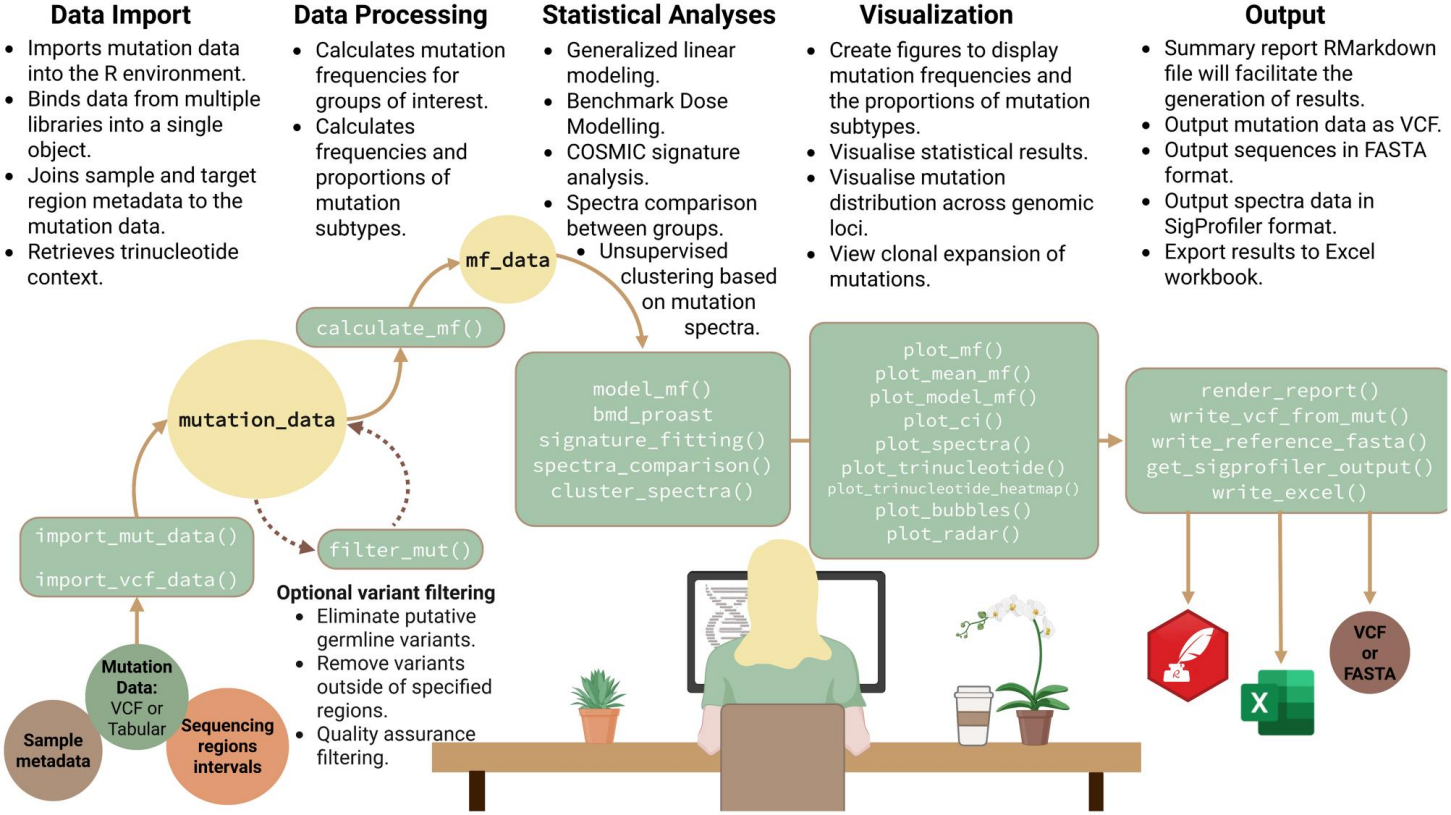
Overview of MutSeqR utilities. Created in Biorender. Dodge A. (2025) https://BioRender.com/zegx6vw

The absence of standardized, transparent methods for ECS data analysis remains a barrier to regulatory implementation, as current studies often use proprietary or inconsistent approaches (Marchetti *et al.* 2023). Before ECS can be used in regulatory risk assessment, steps must be taken to establish standard guidance for analyzing ECS data that will unite the many ECS technologies into a single analytical workflow. To address this, we present MutSeqR, an open source R package for downstream mutation analysis of ECS data (Fig. 1). While MutSeqR does not handle pre-processing of raw sequences or variant calling, it provides robust tools for statistical analysis, integrates tools for characterizing mutational signatures, and generates informative figures. This package aims to enable open source analyses of ECS data, empowering users with flexibility during exploratory analyses while ensuring compatibility across technologies. We demonstrate its utility on two mouse datasets: (i) mouse bone marrow samples sequenced using TwinStrand’s DS, and (ii) mouse liver samples using MutagenTech’s SMM-Seq (single-molecule mutation sequencing). DS sequenced a panel of 48 kb at >10,000× depth. Alternatively, SMM-Seq randomly sequenced genome-wide, trading read depth for breadth, spanning ∼200 Mb. Our analyses highlight how MutSeqR enables detailed, reproducible, and platform-independent assessment of mutagenicity.

## 2 Methods

### 2.1 Datasets

**Figure 2.**
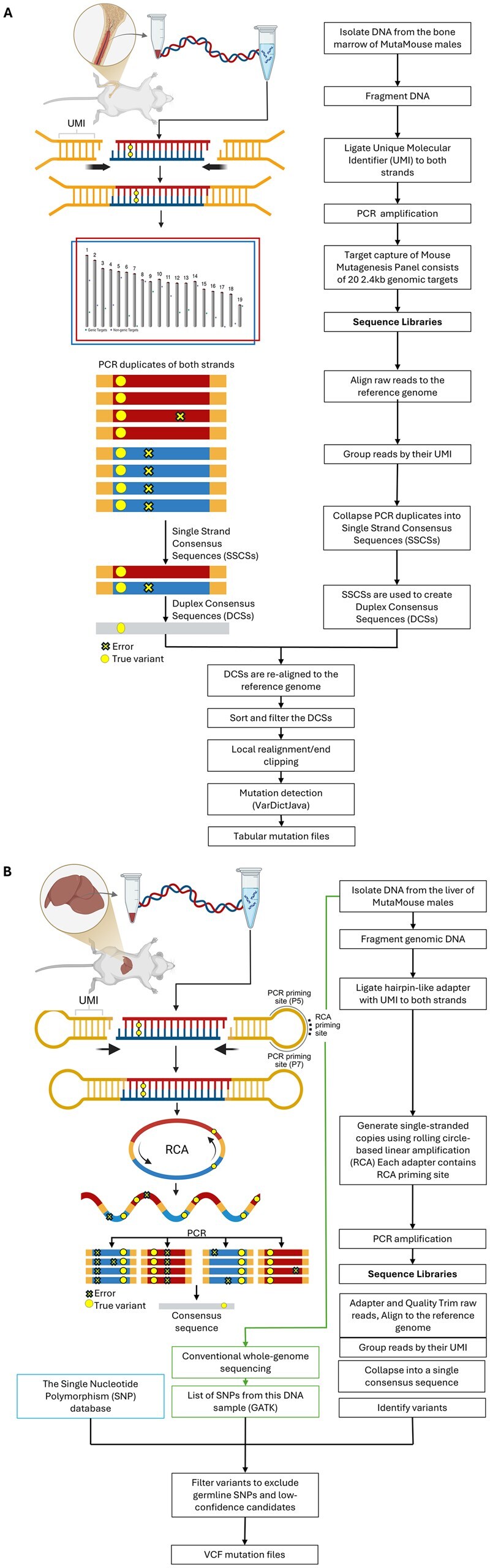
(A) Overview of Duplex Sequencing, adapted from Kennedy *et al.* (2014). (B) Overview of SMM-seq, adapted from Maslov *et al.* (2022). Created in Biorender. Dodge A. (2025) https://BioRender.com/r551hln

Mutation data generated using two ECS technologies were analyzed using MutSeqR to demonstrate its compatibility across platforms. Project 1 included 24 bone marrow DNA samples of MutaMouse males exposed to one of three doses of benzo[a]pyrene (BaP) or a vehicle control for 28 days (LeBlanc *et al.* 2022). DNA samples were sequenced at ≥ 10,000× depth using DS on the Mouse Mutagenesis Panel (https://github.com/twinstrandbio/twinstrandbio-reference-data/), which consists of twenty 2.4 kb target regions (Fig. 2A). Pre-processing of sequencing data was done using TwinStrand’s Mutagenesis App (v. 3.20.1) as described in Valentine *et al.* 2020, to produce tabular mutation data files. Briefly, pre-processing included: raw read alignment, grouping reads by their unique molecular identifiers (UMI) and strand-defining element, quality trimming, error correction of read groups by duplex consensus calling, consensus post-processing, realignment, and variant calling via VarDictJava (Lai *et al.* 2016).

Project 2 comprised 24 liver DNA samples from MutaMouse males following 28-day exposure to one of five doses of benzo[b]fluoranthene (BbF) or a vehicle control (Schuster *et al.* 2024). Samples were subjected to random genome-wide sequencing (∼200 Mb per sample) using SMM-Seq as described (Maslov *et al.* 2022) (Fig. 2B), resulting in VCF files of single-nucleotide variants. Briefly, raw reads are quality-trimmed and aligned to the reference genome. The reads are then grouped by UMI and collapsed into a single consensus sequence. Variants are identified from the consensus sequences, and germline mutations are filtered out.

### 2.2 Data import

Mutation data is imported into R as VCF files or tabular data using the functions *import_vcf_data()* and *import_mut_data()*, respectively. The functions load all files from a user-provided file path and parse them into a unified data frame. VCF files (or block-compressed VCFs) must be single-sample and should conform to the VCF specification (version 4.5; Danecek *et al.* 2011; see required fields in Table 1). We recommend providing a record for every sequenced position (which should include the *total_depth* column), to enable site-specific depth frequency calculations (e.g. subtype frequencies). If non-variant sites are unavailable, users may supply a separate data frame to specify precalculated depths for the desired level of granularity.

**Table 1.** Required fields for mutation data import.

Field	VCF specification	Definition
contig	CHROM	The name of the reference sequence.
start	POS	The start position of the feature. 0-based coordinates are accepted but will be changed to 1-based during import.
end	INFOend	The half-open end position of the feature in the contig.
ref	REF	The reference allele at this position.
alt	ALT	The left-aligned, normalized, alternate allele at this position.
sample	INFOsample | genotype header	A unique identifier for the sample library. For VCF files, this field may be provided in either the INFO field or as the header to the GENOTYPE field.
Optional fields		
alt_depth	VD	The read depth that supports the alternate allele. If not included, the function will assume an *alt_depth* of 1 at variant sites.
total_depth | depth	AD | DP	The total read depth at this position. This field is either *total_depth*, which excludes No-calls (Ns), or *depth*, which includes *o-calls*, if *total_depth* is not available. For VCF files, the *total_depth* is calculated as the sum of *AD*.

Upon import, records are categorized as: (i) no_variant; (ii) snv: single-nucleotide variant; (iii) mnv: multi-nucleotide variant; (iv) insertion; (v) deletion; (vi) complex: REF and ALT are of different lengths and compositions; (vii) sv: structural variants; (viii) ambiguous: IUPAC ambiguity; and, (ix) uncategorized: the variant does not fall into any of the preceding categories (Table 1, available as supplementary data at *Bioinformatics Advances* online). The trinucleotide context for each site is retrieved using *BSgenome*. This includes the reference base plus its two flanking nucleotides. Additional columns are appended to characterize variants (e.g. variant length, variant allele fraction (VAF), GC content). Lastly, SNV subtypes are stratified by pyrimidine reference (C/T) in conjunction with their trinucleotide context, enabling flexible calculation of subtype frequencies. Mutation data can be output as a data frame or a *GRanges* object (Lawrence *et al.* 2013) for downstream analysis.

#### 2.2.1 Application to project datasets

Project 1: Data for each sample was provided as tabular files containing records for all 48, 000 positions on the Mouse Mutagenesis Panel, including *alt_depth* and *total_depth* values. Data was imported using *import_mut_data()*.

Project 2: Data for each sample was provided as compressed VCF files, listing only variant sites. No-variant positions were not included; instead, per-sample total depths were provided separately for mutation frequency calculations. The data was imported using *import_vcf_data()*.

### 2.3 Variant filtering

Following import, mutation data is filtered using *filter_mut()*, which flags variants according to user-specified parameters in the *filter_mut* column. Variants flagged as TRUE in *filter_mut* are automatically excluded from mutation counts in downstream analyses (e.g. in *calculate_mf()*), but are retained in the dataset so their *total_depth* values remain available for frequency calculations. Optionally, filtered records can be removed entirely.

#### 2.3.1 Application to project datasets

Project 1. Filters were applied per TwinStrand’s recommendations:

- *vaf_cutoff = 0.01*. Variants exceeding this VAF were flagged as putative germline variants.
- *snv_in_germ_mnv = TRUE*. SNVs overlapping germline MNVs were flagged as probable artifacts from variant calling. No-calls in reads supporting the germline MNV may create false minor haplotypes from the original MNV that can appear as sub-clonal SNVs.
- *custom_filter = filter*, *custom_filter_val = “EndRepairFillinArtifact”*. A customizable filter was applied to TwinStrand’s column “*filter*”. Variants reported as an End-Repair Fill-in Artifact by TwinStrand’s pipeline were flagged.
- *regions = “TSpanel_mouse”*. Records outside target regions were removed. The regions for TwinStrand’s Mouse Mutagenesis Panel are stored in the package files. Upon specification, records that are >1bp must start and end within the target region or be removed.
- *rm_filtered_mut_from_depth = TRUE*. For flagged variants (other than putative germline), *alt_depth* was subtracted from *total_depth*, treating these as No-calls.

Project 2. MutagenTech performed variant filtering. Variants were filtered against the single-nucleotide polymorphisms database (dbSNP) to remove known mouse variants, and additional germline variants were removed based on matched whole-genome sequencing at 20× depth.

### 2.4 Calculating mutation frequencies

Mutation frequency (MF) is calculated by dividing the sum of mutations by the sum of the *total_depth* (mutations/bp) using the function *calculate_mf()*. Users can supply grouping variables (e.g. by sample, experimental group, genomic region, subtype), allowing MF to be computed across various categories. To prevent double-counting read depth when multiple variants are called at the sample position in a sample, only the first record retains its *total_depth*, while subsequent records are set to zero. For datasets lacking per-site depth, precalculated depths can be provided via the *precalc_depth_data* parameter.

Two mutation counting methods are implemented (Dodge *et al.* 2023):

- The Minimum Independent Mutation Counting Method (Min): Each mutation is counted once, regardless of the number of reads supporting the non-reference allele. This method assumes that multiple instances of the same mutation within a sample are the result of a single mutational event that underwent subsequent clonal expansion.
- The Maximum Independent Mutation Counting Method (Max): Multiple identical mutations at the same position within a sample are counted as independent mutational events. All reads with the non-reference allele are included in the count.

MutSeqR automatically calculates both Min and Max mutation frequencies (MF_Min_ and MF_Max_).

### 2.5 Mutation subtypes: frequencies and proportions

*calculate_mf()* can also group mutations by their subtype at various resolutions. All mutations can be resolved to their variation type, and SNVs can be further resolved into: (i) 6-base: SNV substitution types (subtypes) reported in their pyrimidine reference; (ii) 12-base: SNV subtypes; (iii) 96-base: 6-base SNV subtypes reported within their trinucleotide context; and (iv) 192-base: 12-base SNV subtypes reported within their trinucleotide context. Mutations for each subtype are summed across groups. The *total_depth* is summed across groups for positions at which the subtype can occur. In the simplest example, for the 6-base SNV subtypes, the two possible reference bases are C or T; hence, the depth is calculated separately for C: G positions and T: A positions. The function also calculates the proportion of mutations for each subtype, normalized to read depth and coverage of those reads. The normalized proportion of a subtype is calculated by dividing the MF of the given subtype by the sum of all subtype frequencies in a group.


(1) Ps=Ms/Ds∑sMs/Ds 

*P*_s_ Mutation proportion for subtype s, normalized to the read depth. *M*_s_ mutation count for the subtype s. *D*_s_ read depth for s context.

If per-site depth is unavailable, users can supply precalculated depth at the chosen subtype resolution. Per our previous example, users would supply precalculated depth for all C: G positions and T: A positions in each group. When this is not possible, (non-normalized) subtype proportions (*P*′_s_) are reported by dividing subtype mutation counts (*M_s_*) by the total number of mutations in the group (*M*_total_).


(2) P's=Ms/Mtotal

#### 2.5.1 Application to project datasets

Project 1: Per-site *total_depth* values were summed to calculate MF and normalized subtype proportions.

Project 2: Precalculated *total_depth* values provided by MutagenTech were used for MF calculation.

### 2.6 Statistical approaches

#### 2.6.1 Mutation frequency modelling

The *model_mf()* function quantitatively assesses how MF varies with experimental factors. It models the proportion of mutated reads as a function of user-defined fixed and/or random effects and interaction parameters. Depending on the supplied effects, *model_mf()* will automatically choose to fit either a generalized linear model (GLM) or a generalized linear mixed model (GLMM). Mutation counts are treated as binomially distributed, given (i) there is a finite number of sequenced bases, (ii) a mutation at any given base is equally probable (acknowledging this does not fully reflect biological reality), and (iii) mutations occur independently of other mutations. To handle over-dispersion, GLM fits a quasibinomial distribution, unless dispersion <1, in which case a binomial distribution is used; GLMMs employ a binomial distribution throughout. *model_mf()* provides estimates of the mean for each level of the fixed effects. Furthermore, pairwise comparisons can be performed based on a user-supplied table. Mean estimates and comparisons are conducted using the doBy R library (Halekoh and Højsgaard 2025). The *esticon()* function is used to generate estimates for specified linear combinations of the model parameters based on the fitted model:


(3) g(μi)=logit(μi)=log⁡μi1-μi =Xiβ

where $\mu_{i}$ is the expected value of the response for group *i*, $X_{i}$ is the *i*th column of the design matrix for the fixed effects, and ***β*** is the vector of coefficients associated with the fixed effects.

This formula can be rewritten such that:


(4) μi=exp⁡(Xiβ)1+exp⁡(Xiβ)≈exp⁡(Xiβ)=MFi

Since the mutation frequencies (${\mathrm{MF}}_{i}$) are assumed to be small (∼10^−8^),


(5) 1+exp⁡(Xiβ)≈1

The linear combination of the model parameters would then be:


(6) θ^=cTβ^

where $c^{T}$ would represent the contrast vector specifying the weights, e.g. if the model includes two treatment groups with estimated coefficients ${\hat{\beta }}_{1}$ and ${\hat{\beta }}_{2}$, the contrast or linear combination to estimate the difference would be:


(7) c=(0, 1, -1, 0,…, 0)

resulting in


(8) θ^=cTβ^=β^1-β^2

Since the mutation frequencies are small, exponentiating $\hat{\theta }$ would approximate the fold change of group 1 to group 2. In the case of a log-link (common for rare event models like Poisson or binomial GLMs with small probabilities), this provides a direct estimate of the relative risk or the rate ratio between groups.

Estimates of the back-transformed standard errors are approximated using the delta method. The *P* values are adjusted for multiple comparisons using the Sidak method. These methods are based on approaches developed for the Transgenic Gene Mutation Reporter (TGR) assay (Fung *et al.* 1998).

##### 2.6.1.1 Application to project datasets

We used *model_mf()* to assess the effect of BaP (Project 1) and BbF (Project 2) dose on MF_Min_ using a GLM with quasibinomial dispersion. For Project 1, we extended this model to a GLMM using the effects of dose and sequencing target with the sample as a random effect.

#### 2.6.2 Benchmark dose modelling

Dose–response models are essential for quantitative risk assessment of mutagenicity, as they provide a framework to evaluate the levels at which exposure to a substance might cause an adverse effect. The benchmark dose (BMD) is a dose that produces a predetermined change in the measured response, defined as the benchmark response (BMR). The BMD is used as a point of departure to derive human health-based guidance values to inform regulatory risk assessment. *bmd_proast()* runs a parameterized version of the proast71.1 R package (www.rivm.nl/en/proast), a widely used software designed to be consistent with methods used by regulatory authorities (More *et al.* 2022). This function analyses continuous, individual MF data following a log transformation using exponential, Hill, inverse exponential, and log-normal models, selecting the best fit by AIC. Confidence intervals are computed via profile likelihood or bootstrap model averaging (recommended). MutSeqR calculates the model-averaged BMD as the median BMD of all bootstraps. BMR values may be specified as a relative increase or as one standard deviation from the control. Covariates are supported.

##### 2.6.2.1 *Application to project datasets*

We used *bmd_proast()* to perform BMD analysis with model averaging to calculate the BaP/BbF dose that produces a 50% relative increase in MF_Min_ compared to control (White *et al.* 2020) with 90% confidence intervals.

#### 2.6.3 Comparing mutation spectra

A mutation spectrum is the pattern of mutation subtypes within a sample or group, which can reveal mutagenic mechanisms associated with endogenous processes or chemical exposure. *spectra_comparison()* constructs an *R*∗*T* contingency table of mutation counts where *R* is the number of subtypes and *T* represents the two groups being compared (Piegorsch and Bailer 1994). It then tests for differences in subtype proportions between groups using the *G*^2^ likelihood ratio statistic.


(9) G2=2∑i=1R∑j=1TYijlog⁡(YijEij)

where $Y_{ij}$ is the observed mutation count and $E_{ij}$ the expected counts under the null hypothesis of no difference. For large sample sizes, *G*^2^ is referred to a *X*^2^ distribution with (*R*−1)(*T*−1) degrees of freedom. However, when *N*/(*R* − 1) < 20 (where *N* is the total mutation count across groups), an F-distribution is used for greater reliability, reducing false positives when sample sizes are small.

Spectrum analysis assumes independence among the observations. This is appropriate for mutations from mixed populations, but not for those derived from a single progenitor cell. Accordingly, the MF_Min_ method is used here to ensure counts are independent.

##### 2.6.3.1 Application to project datasets

Mutation spectra were compared between BaP/BbF dose groups and controls at multiple subtype resolutions.

#### 2.6.4 Hierarchical clustering of mutation spectra

Hierarchical clustering groups similar data points into nested clusters based on pairwise similarities (Everitt 1974, Hartigan 1975). *cluster_spectra()* clusters samples (or user-defined groups) according to their mutation spectra, which may be represented as subtype counts, frequencies, or proportions. MutSeqR computes distance using *dist()* from the stats library (default Euclidean). The resulting distance matrix is then passed to *hclust()* to cluster samples using the specified linkage method (default Ward). Optimal leaf ordering is performed with the dendsort library, producing a dendrogram of sample relationships.

##### 2.6.4.1 Application to project datasets

For each project, samples were clustered by the proportions of their 6-base SNV subtypes and non-SNV variation types using default parameters.

### 2.7 Mutational signature assignment

Mutational processes generate characteristic patterns of mutations, known as mutational signatures. Distinct mutational signatures have been extracted from various cancer types and normal tissues using data from the Catalogue of Somatic Mutations in Cancer (COSMIC) database and compiled into a known collection (https://cancer.sanger.ac.uk/signatures/). Signatures have also been characterized for specific environmental chemicals (Kucab *et al.* 2019; https://signal.mutationalsignatures.com/), germline mutations (Seplyarskiy *et al.* 2021), and DNA repair deficiencies (Zou *et al.* 2021). Linking ECS mutational profiles of specific mutagens to existing mutational signatures provides empirical evidence for the contribution of environmental mutagens to the mutations found in human cancers and informs on mutagenic mechanisms.

MutSeqR’s *signature_fitting()* utilizes the SigProfiler suite of tools (Díaz-Gay *et al.* 2023, Khandekar *et al.* 2023) to assign SBS signatures from the COSMIC database to the 96-base SNV subtypes of a given group by creating a virtual environment to run Python using reticulate. Thus, the *signature_fitting()* function facilitates interoperability between these tools for users less familiar with Python and assists users by coercing the mutation data to the necessary structure for the SigProfiler tools. SNVs in their 96-base trinucleotide context are summed across groups to create a mutation count matrix by SigProfilerMatrixGenerator (Khandekar *et al.* 2023). *Analyze.cosmic_fit* (SigProfilerAssignment; Díaz-Gay *et al.* 2023) is then run to assign mutational signatures to each group using refitting methods, which quantifies the contribution of a set of signatures to the group’s mutational profile. Cosine similarity values are generated to compare the reconstructed mutational profile to the original mutational profile of the group, with values >0.9 indicating a robust reconstruction.

#### 2.7.1 Application to project datasets

Mutations were grouped by BaP/BbF dose group for signature assignment

### 2.8 Data visualization

MutSeqR has several plotting functions for data visualization.

**Figure 3.**
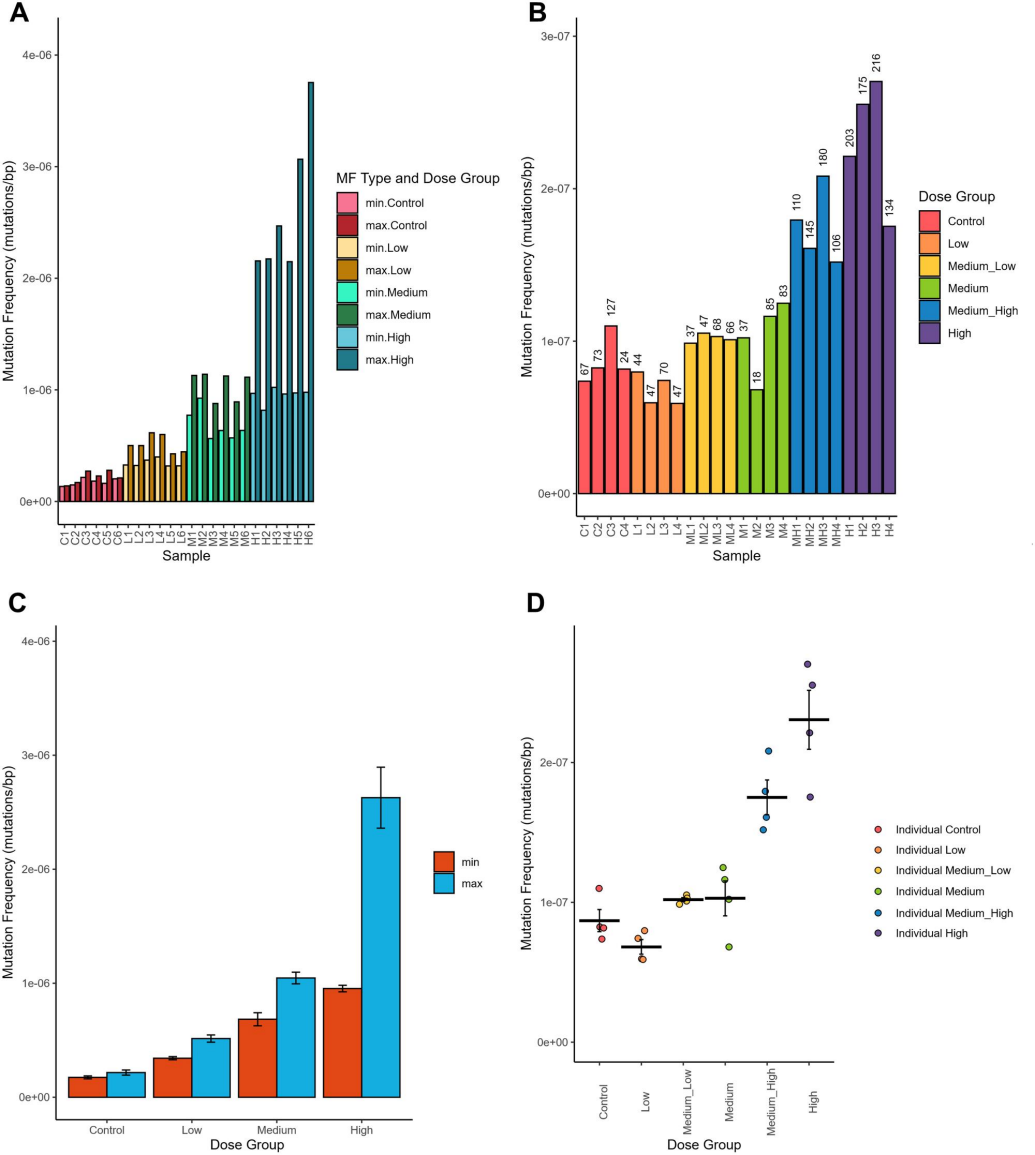
(A) Duplex Sequencing mutation frequencies (MF) in the bone marrow of MutaMouse males at four doses of BaP. Bars represent the MF (mutations per bp) for each animal. Bars are coloured based on BaP dose group and MF type (Min or Max). Plotted using *plot_mf()*. (B) SMM-seq MF in MutaMouse males’ liver at six BbF doses. Data labels indicate the total mutation count per animal. (C) Mean Duplex Sequencing MF per BaP dose group. Bars are coloured based on MF type. Error bars reflect the SEM. Plotted using *plot_mean_mf()*. (D) Mean SMM-seq MF per BbF dose group. Lines represent the mean MF, while the points represent individual MF values per animal and are coloured based on BbF dose group. Error bars represent the SEM. Plotted with *plot_mean_mf()*.

**Figure 4.**
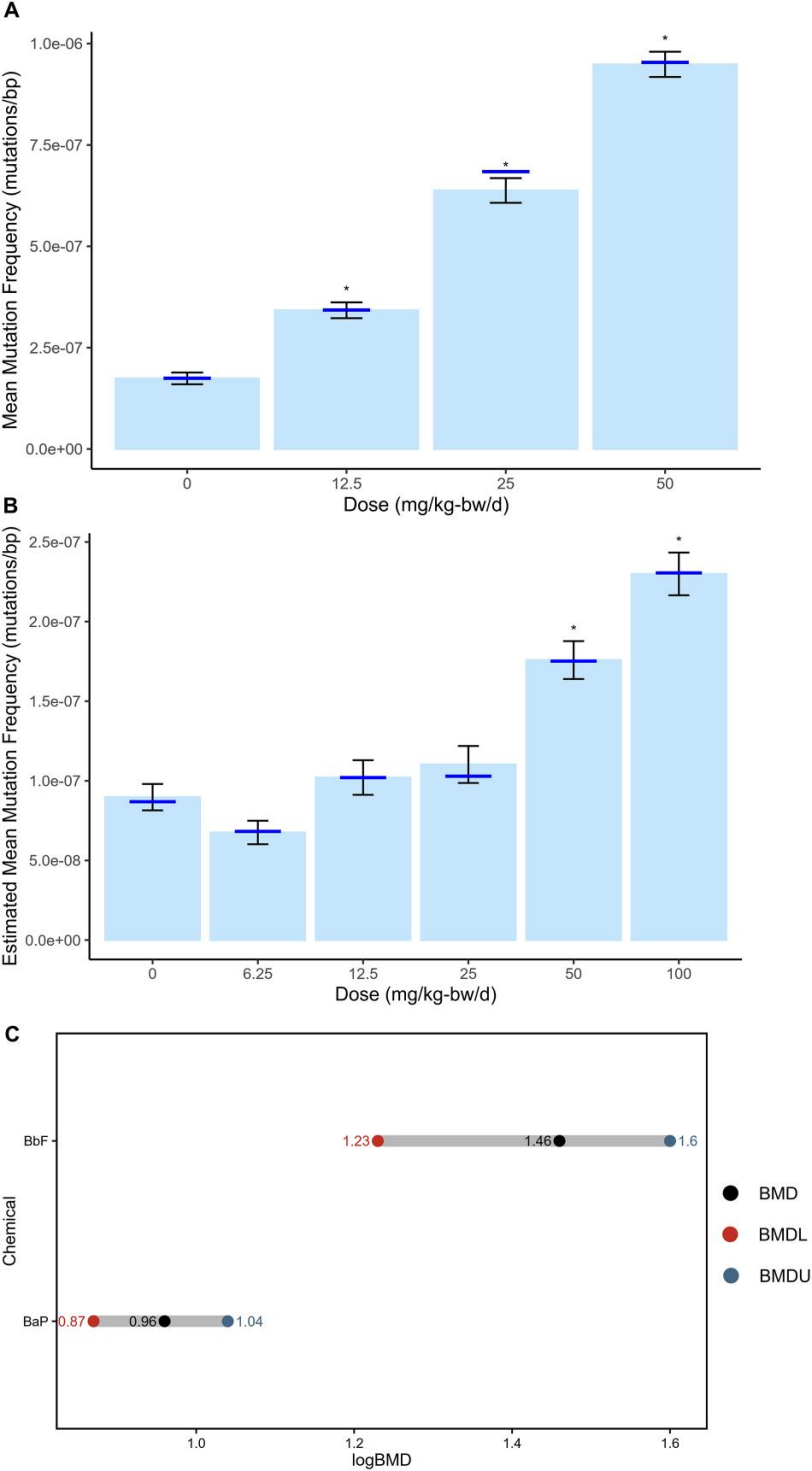
GLM results. Light blue bars represent the model-estimated mean mutation frequency minimum (MF_Min_) for (A) BaP and (B) BbF dose groups. Error bars represent the estimated SEM. Asterisk indicates a significant increase in MF_Min_ relative to control (*model_mf()* GLM, *P < *.05). Plotted using *plot_model_mf()*. Dark blue bars representing the empirical mean MF_Min_ were added using ggplot2. (B) Benchmark dose with 90% confidence intervals representing the dose at which a 50% increase in MF_Min_ from controls occurs for each chemical, calculated using *bmd_proast()*. Black points represent the BMD, red points represent the BMDL, and blue points represent the BMDU. Plotted using *plot_ci()*.

**Figure 5.**
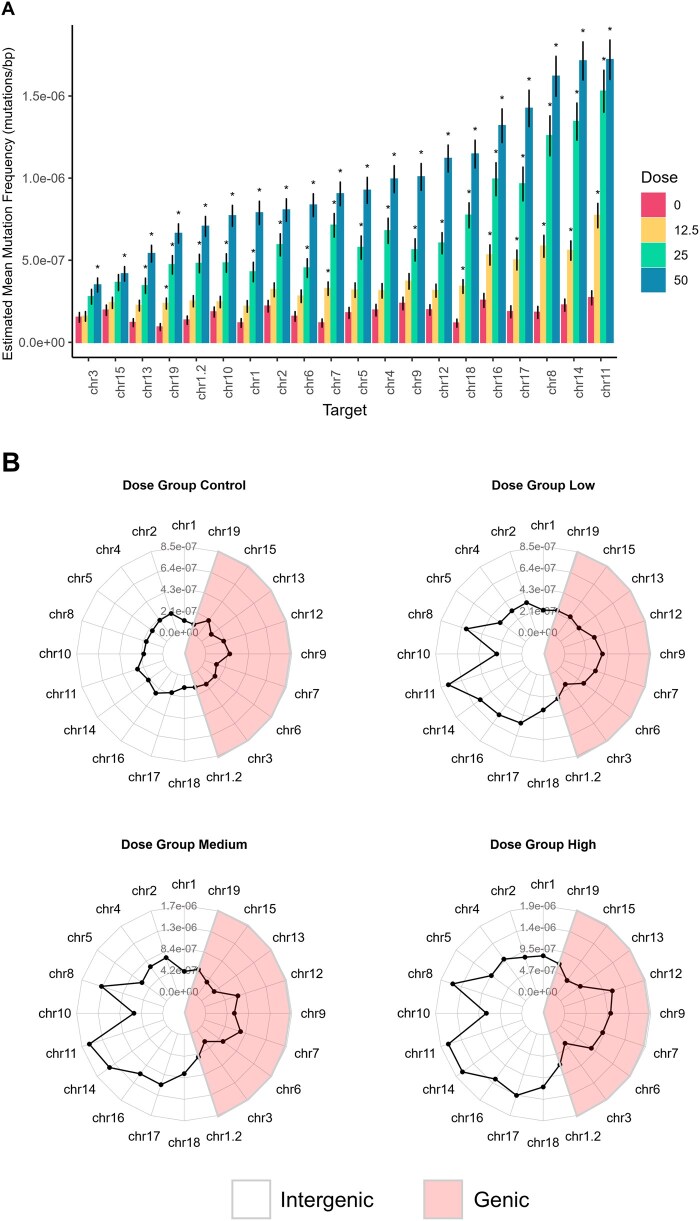
(A) Spontaneous and BaP-induced MF_Min_ by Duplex Sequencing target, ordered from lowest MF_Min_ in the high BaP dose group to the highest. Data are estimated mean MF_Min_ ± SEM mutation per bp, for each target, separated and coloured by dose group. Asterisks indicate a significant increase in MF_Min_ compared to the control (*model_mf();* GLMM, *P < *.05). Plotted using *plot_model_mf()*. (B) Mean MF_Min_ per Duplex Sequencing target, facetted by dose group. Duplex Sequencing targets are organized based on genic status; targets within genic regions are highlighted in red, targets within intergenic regions are in white. Plotted with *plot_radar()*. Red shading and genic status legend added separately.

**Figure 6.**
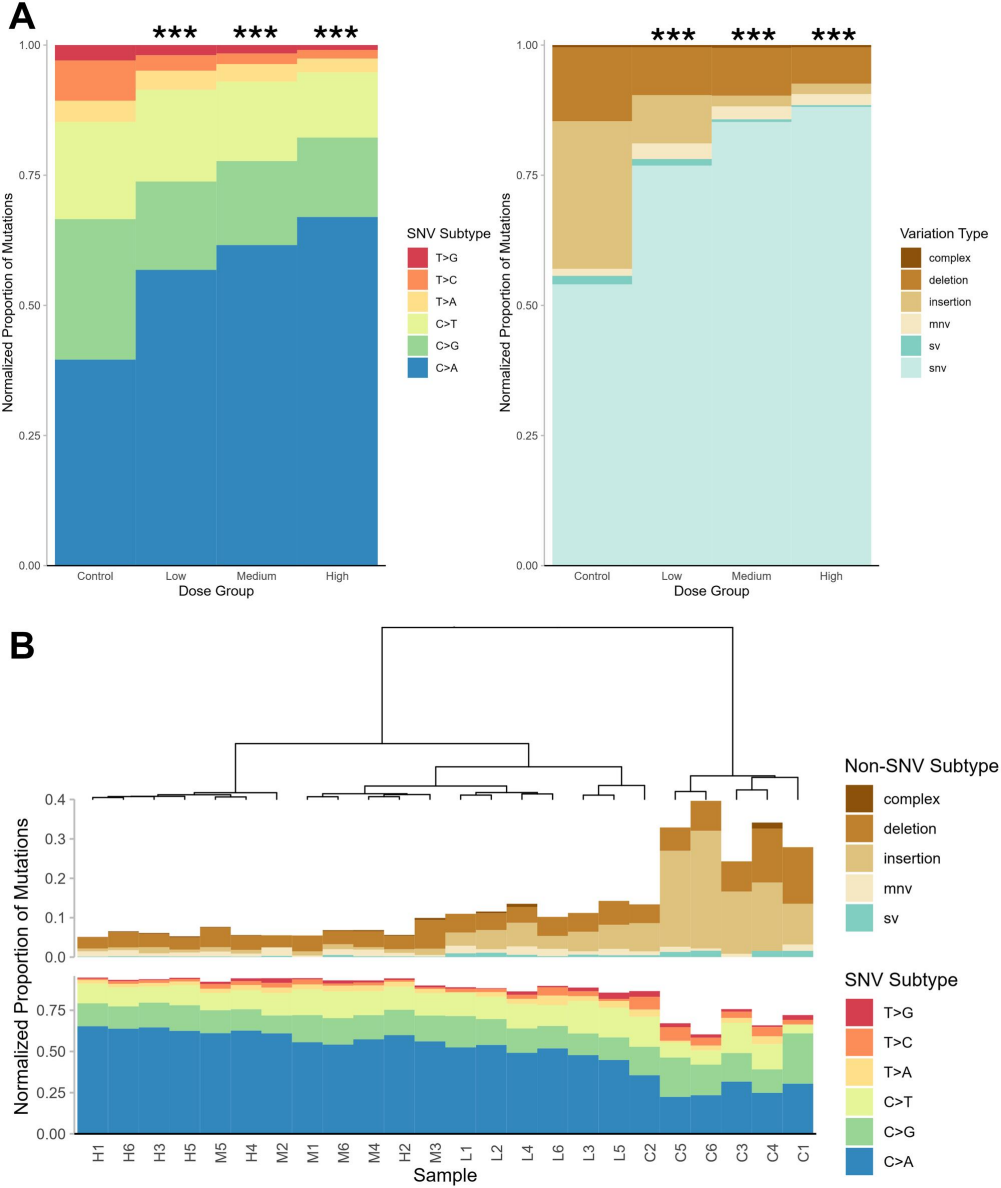
(A) Mutation spectrum of control and BaP dose groups measured by Duplex Sequencing in the bone marrow of MutaMouse males. Normalized proportions of mutation subtypes are represented by colour within the stacked bar for each BaP dose group. Left is the proportion of SNV subtypes out of total SNVs, right is the proportion of variation types out of total mutations. For each panel, asterisks indicate a significant difference in the mutation spectra compared to the control (*spectra_comparison()*; modified contingency table, *P < *.05). Plotted using *plot_spectra()*, asterisks added separately. (B) Mutation spectrum of individual animals measured by Duplex Sequencing. Animals are clustered into groups based on the Euclidean distance between their subtype proportions using *cluster_spectra()*. Proportions are SNV subtypes (bottom) and non-SNVs (top) by total mutations. All proportions are normalized to their given reference depth. Sample identifiers (x-axis) denote dose group: C—control, L—low, M—medium, H—high. Plotted with *plot_spectra()*.

**Figure 7.**
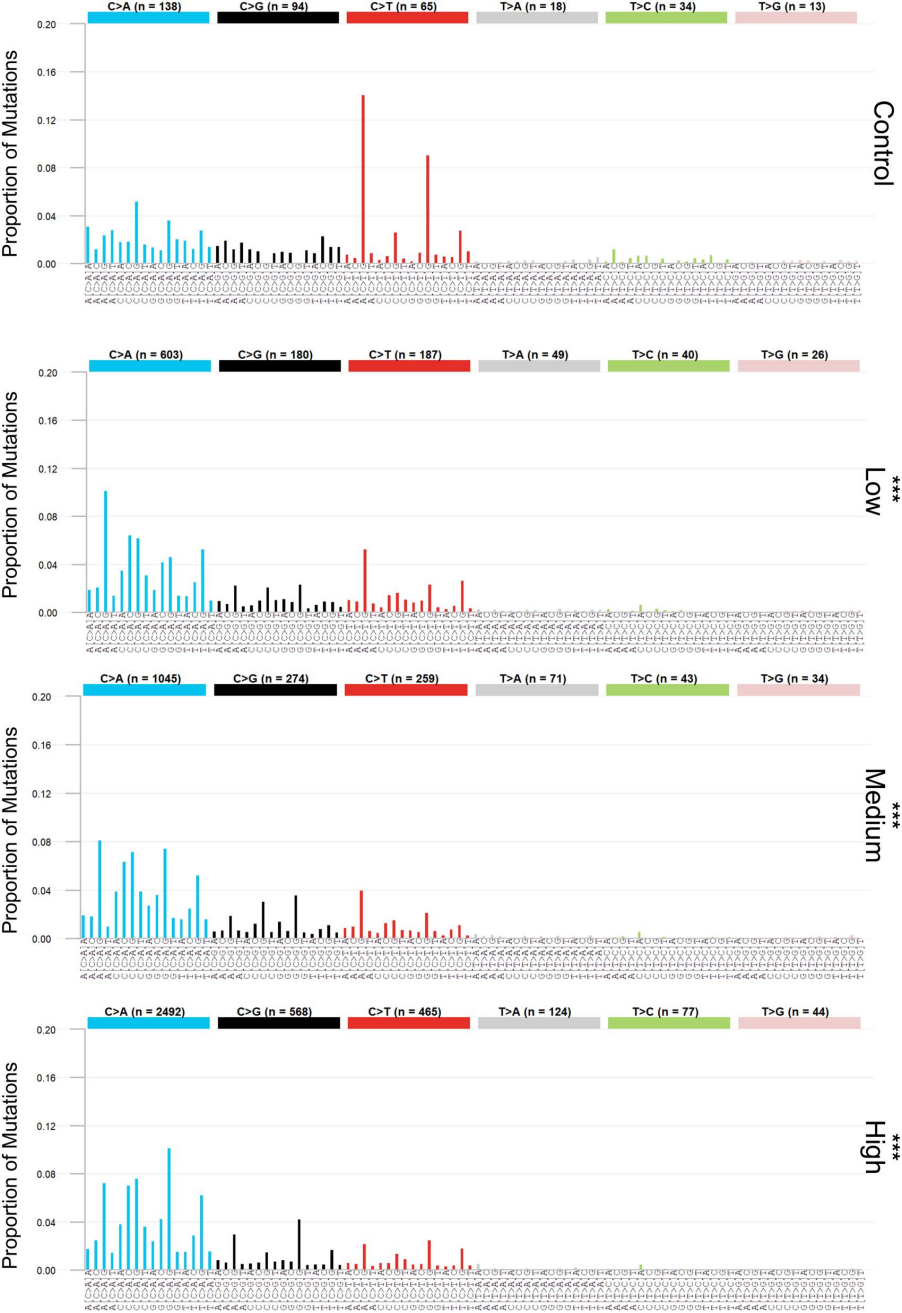
Normalized proportion of mutation subtypes within their 96-trinucleotide context for control and BaP dose groups, measured by Duplex Sequencing. Bars are coloured based on the normalized SNV subtype. Data labels represent the number of mutations for each normalized SNV subtype within that dose group. Asterisks indicate a significant difference in the trinucleotide mutation spectra relative to the control (*spectra_comparison()*; modified contingency table, *P < *.05). Plotted using *plot_trinucleotide()*; dose group labels and asterisks were added separately.

**Figure 8.**
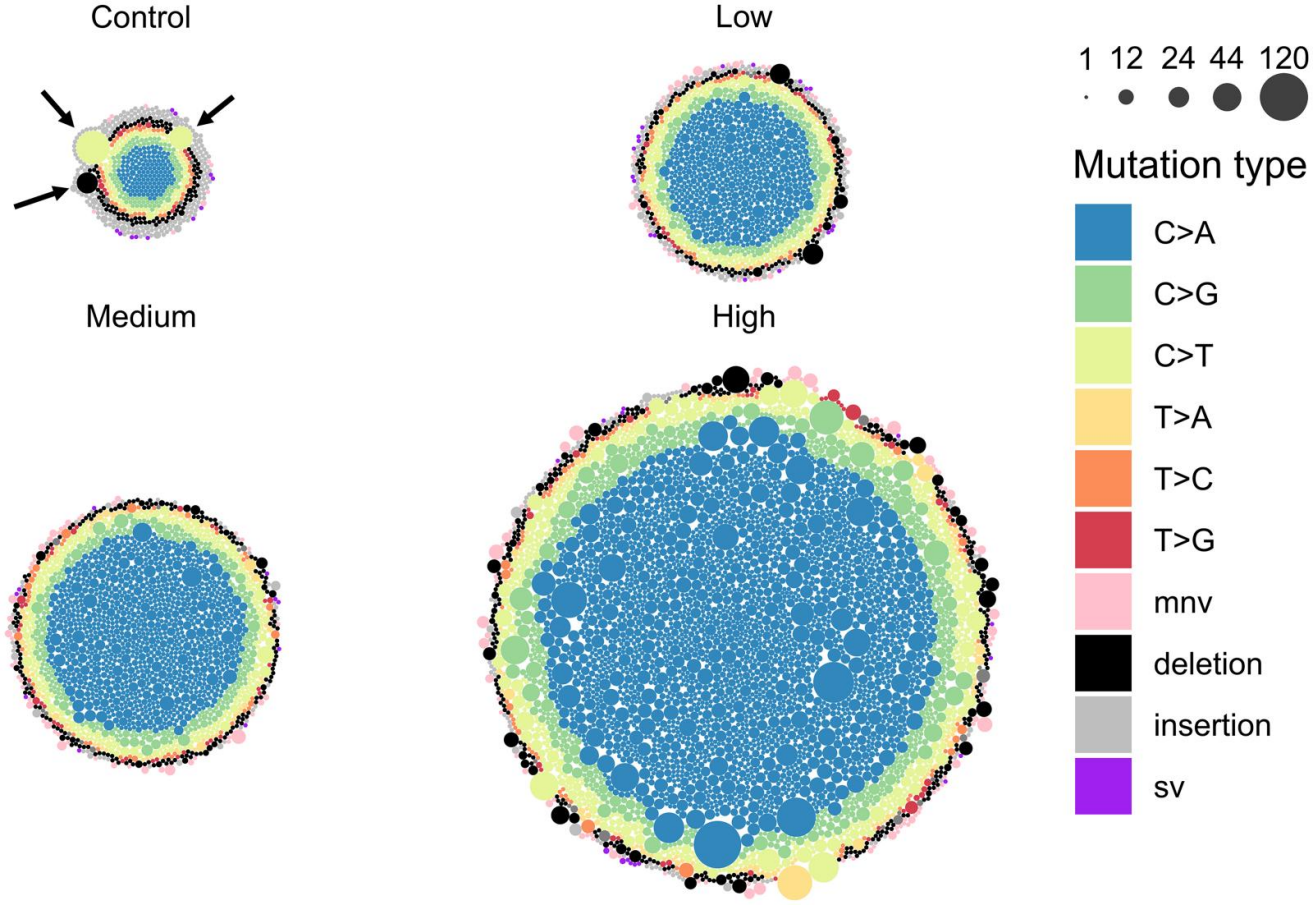
Multiplet mutations measured by Duplex Sequencing for control and BaP-treated groups in MutaMouse bone marrow. Each circle represents a mutation, coloured by mutation subtype. The size of the circle is scaled by the mutation’s alternative depth. Arrows indicate three multiplets as examples in the control group. Plotted using *plot_bubbles()*. Arrows added separately.

1. *plot_mf()*/*plot_mean_mf()* creates a plot of the MF or mean MF from *calculate_mf()* output (Fig. 3). Variable plotting aesthetics (bars, points, lines) are offered. MF_Min_ and MF_Max_ can be displayed individually, side by side, or stacked. Data can be coloured based on a specified variable and labelled with mutation count or frequency. The standard error of the mean may be shown as error bars. Individual samples may be plotted alongside the mean values.
2. *plot_model_mf()* plots the point estimates derived using *model_mf()* (Figs 4A and 5A). Data is presented as either bars or points. Users can include the estimated standard error as error bars and add significance labels based on pairwise comparisons.
3. *plot_ci()* creates a plot of BMD confidence intervals for easy comparison between responses (Fig. 4B).
4. *plot_spectra()* creates a transition-transversion plot of mutation spectra for user-defined groups at the desired subtype resolution (Fig. 6A). Data can represent subtype mutation count, MF, or proportion. This function can call upon *cluster_spectra()* to overlay a dendrogram of the clustered groups (Fig. 6B).
5. *plot_trinucleotide()* creates bar plots of the 96-base SNV (trinucleotide) spectrum for all levels of a user-defined group (Fig. 7). Data can represent subtype mutation count, MF, or proportion. Aesthetics are consistent with COSMIC trinucleotide plots.
6. *plot_trinucleotide_heatmap()* creates a heatmap plot of 96-base SNV proportions. Plots can be facetted by a specified grouping variable in the MF data.
7. *plot_radar()* generates a radar/spider plot of MF  given a user-defined group (Fig. 5B). Data may be plotted on a single plot or facetted by group.
8. *plot_bubbles()* Creates a bubble plot with circles representing each mutation in a given group (Fig. 8). Plots are facetted by group. Circle size can reflect either *alt_depth* or VAF. Circles may be coloured by a specified variable in the mutation data (e.g. subtype/loci).

## 3 Results

### 3.1 Variant filtering

Project 1: *filter_mut()* flagged or removed 2660 out of 1 152 911 rows of mutation data. About 612 variants were identified as putatively germline, and 20 SNVs were flagged because they overlapped with germline MNVs; 2021 variants were flagged by the custom filter; 22 rows were removed because they extended outside of the target regions.

### 3.2 Quantifying mutation frequencies

MutSeqR’s *calculate_mf()* provided MF per sample for both projects, and results were visualized as a bar plot using *plot_mf().*  Figure 3A and B displays MF per sample for Projects 1 and 2, respectively. Bars were coloured and ordered by dose. Optional data labels were added to Fig. 3B, displaying the mutation count per sample. These plots offer a useful first exploration of the mutation data. Project 1 shows that BaP causes a dose-dependent increase in MF_Min_ and MF_Max_, with low inter-sample variability, and a high number of recurrent mutations at high doses. In Project 2, MF_Min_ increases with BbF dose. As SMM-Seq sequenced each site only once, MF_Max_ could not be assessed.

Mean ± SEM MF per dose was calculated and visualized using *plot_mean_mf()*. Figure 3C displays Project 1 mean MF_Min_ and MF_Max_ as a bar plot with bars coloured by MF type. Under the Min assumption, we calculated a mean of 17.4 ± 2.26, 34.3 ± 1.38, 68.4 ± 5.72, and 95.4 ± 2.87 (×10^−8^ mutations/bp) for the control, low, medium, and high doses, respectively. Under MF_Max_, a mean of 21.7 ± 2.26, 51.5 ± 3.18, 105 ± 5.11, and 263 ± 26.7 (×10^−8^ mutations/bp) was calculated, respectively. Figure 3D displays Project 2 mean MF_Min_ as a line alongside the individual values for each sample, demonstrating the flexible aesthetics of the plotting function. Mean MF_Min_ (×10^−8^ mutations/bp) was 8.70 ± 0.792, 6.82 ± 0.521, 10.2 ± 0.141, 10.3 ± 1.25, 17.5 ± 1.24, and 23.1 ± 2.11 for the control, low, medium-low, medium, medium-high, and high doses, respectively.

### 3.3 Modelling dose effects

The dose-dependent effects of BaP/BbF on MF_Min_ were quantified using two methods. First, a GLM was fit to estimate the dose effect on the MF_Min_ using *model_mf()*. This model assumes that the response variable follows a specified distribution (e.g. an over-dispersed binomial) and uses a link function to relate the mean response to the predictors. To assess model fit, Pearson residuals were visualized using a histogram (Fig. S1A and C, available as supplementary data at *Bioinformatics Advances* online) and a Q–Q plot (Fig. 1B and D, available as supplementary data at *Bioinformatics Advances* online) to check for deviations from the model assumptions. These plots were automatically generated by *model_mf()*. BaP induced a significant increase in MF_Min_ for all dose groups, reaching a maximum of a 5.5-fold increase at the high dose (Fig. 4A). BbF induced a significant increase in MF_Min_ for the top two dose groups, reaching a maximum of a 2.6-fold-increase at the high dose (Fig. 4B). Model results were plotted using *plot_model_mf()*, which created a bar plot of the model-estimated mean MF per dose. Significance labels were added based on the specified comparisons. The output is a *ggplot* object that is easily modifiable using ggplot2 functions. In this way, we included the empirical mean MF_Min_ as dark blue lines. Estimated mean values were almost identical to empirical values for all BaP and BbF doses.

Next, BMD models were used to analyse the dose–response. We used *bmd_proast()* with model averaging to calculate the lower (BMDL) and upper (BMDU) 90% BMD confidence intervals. We estimated that a BaP dose of 9.11 mg/kg-bw/d (BMDL = 7.38, BMDU = 10.9) induces a 50% relative increase in MF_Min_ from control (Table 2, available as supplementary data at *Bioinformatics Advances* online). An estimated BbF dose of 28.8 mg/kg-bw/d (BMDL = 17.0, BMDU = 39.4) induces a 50% relative increase in MF_Min_ from control (Table 3, available as supplementary data at *Bioinformatics Advances* online). The BMD_50_ and their confidence intervals were plotted using *plot_ci()* (Fig. 4C).

### 3.4 Mutation spectra

MutSeqR’s *calculate_mf()* was used to resolve mutations to their variation types. SNVs were examined at both the 6-base and 96-base resolutions and visualized for each dose using *plot_spectra()*. BaP induced primarily SNVs, followed by small deletions, small insertions, MNVs, and lastly SVs (Fig. 6A). *spectra_comparison()* revealed that the BaP subtype proportions were significantly different from the controls at all doses (*P < .*001), indicating that BaP induces a unique pattern of mutation subtypes. Specifically, we observed dose-dependent increases in the proportions of C: G > A: T mutations. This is consistent with the known mode of action of BaP, which forms bulky DNA adducts mostly at the N2 of guanine through its metabolite benzo(a)pyrene-7,8-diol-9,10-epoxide (Beal *et al.* 2015, Liamin *et al.* 2017, LeBlanc *et al.* 2022). We next used *cluster_spectra()* to cluster the samples based on their subtype proportions (Fig. 6B). Samples from the same dose groups largely clustered together. Clear distinctions were made between the high and medium doses versus the control and low doses. Finally, we plotted the proportions of the 96-base spectra for each dose using *plot_trinucleotide()* (Fig. 7). We observed that high proportions of C: G > T: A mutations in the control were specifically associated with CpG sites. These proportions declined with increasing doses of BaP, favouring C: G > A: T mutations at CpG sites.

SMM-Seq recorded only SNV mutations for Project 2. BbF induced primarily C: T > A: G mutations (Fig. 2A, available as supplementary data at *Bioinformatics Advances* online). *spectra_comparison()* revealed that the BbF subtype proportions were significantly different from the controls at the top three doses (*P < .*001) at the 6-base resolution. Thus, despite not inducing a significant increase in MF_Min_, BbF causes significant shifts in the mutation spectrum at the medium dose. Specifically, we observed dose-dependent increases in the proportions of C: G > A: T mutations. Following clustering, distinct sample groups were made between the high, medium-high, and medium dose groups versus the control, low and medium-low dose groups (Fig. 2B, available as supplementary data at *Bioinformatics Advances* online). Finally, *plot_trinucleotide()* revealed increases in the C: G > A: T mutations within the CCA, CCC, and CCT context at the medium-high and high-dose groups (Fig. 3, available as supplementary data at *Bioinformatics Advances* online, shows Control, Medium-High, and High dose groups).

### 3.5 Signature assignment

We next compared the 96-base spectra to the SBS signatures of the COSMIC database using *signature_fitting()*. The cosine similarity between the reconstructed profile and the spectra was high (>0.9) for all dose groups except for the BbF medium-low dose (0.855). Within the BaP groups, we observed a dose-dependent increase in the contribution of SBS4 (97.51% at high dose), a signature found in lung cancers attributed to tobacco smoking. BaP is prevalent in tobacco smoke (Adesina *et al.* 2022); thus, we expect its mutation spectra to be associated with tobacco-smoke mutational signatures. Additional signatures with minor association with our BaP groups were SBS1 and SBS5, each associated with aging, and SBS94, whose aetiology is unknown. Similarly, SBS 4 was the prevalent signature in the BbF medium-high and high dose groups at 63% and 66%, respectively. BbF is also prevalent in tobacco smoke; thus, this result is aligned with expectations. Additional signatures found included SBS 5 and SBS 1, SBS 58—a known sequencing artifact, and SBS 3, associated with Azathioprine treatment. Plots depicting the original mutation spectra, the reconstructed profile, the contributing signatures and the solution statistics were automatically generated for each group by *signature_fitting()* for BaP (Fig. 4, available as supplementary data at *Bioinformatics Advances* online) and BbF (Fig. 5, available as supplementary data at *Bioinformatics Advances* online, control, medium-high, and high dose groups shown).

### 3.6 Region analysis

Analyzing the individual responses of the 20 DS targets provides clues on inter-locus variability in mutation susceptibility. These regions were supplied to *import_mut_data()*, which annotated sites with their targets based on position. MutSeqR then calculated the per-sample MF for each target with *calculate_mf()*. This was then supplied to *model_mf()*, which ran a GLMM that evaluated the effects of target and dose on the MF_Min_. Residuals were assessed (Fig. 6, available as supplementary data at *Bioinformatics Advances* online). We specified comparisons between the BaP doses and the control for each target, revealing a significant increase in MF_Min_ in the high-dose group compared to the control for all targets. All targets except for those on chr3 and chr15 were significantly increased at the medium dose, and eight of the 20 targets were significantly increased at the low dose. The model-estimated mean MF_Min_ was plotted using *plot_model_mf()* (Fig. 5A). This function can plot model results with up to two fixed effects. Here, we used target as the x-axis and dose as the colour. The x-axis order was defined as targets ascending by the MF_Min_ value at the high dose. Significance labels were added based on the specified comparisons. Our visualization of the data revealed that MF_Min_ varied extensively between targets, with this effect exacerbated by dose. Within the control, there was a three-fold difference between the target with the highest MF (chr11) and the one with the lowest (chr19). Within the high-dose group, there was a five-fold difference in MF_Min_ between the target with the highest MF (chr11) and the one with the lowest MF (chr3). Finally, we plotted the mean MF_Min_ per target for each dose using *plot_radar()*. Targets were organized based on their genic context. This showed a trend for higher MF_Min_ in intergenic targets compared to genic targets at all dose groups (Fig. 5B).

### 3.7 Visualization of multiplets

In Project 1, we observed considerable inflation of the MF_Max_ compared to the MF_Min_. However, from an initial analysis, it was unclear whether the increased MF_Max_ was a result of a few highly recurrent mutations or several moderately recurrent mutations. We used *plot_bubbles()* to visualize the *alt_depth* of every mutation called for each dose (Fig. 8). Bubble plots allow us to analyse the distribution and density of multiplet mutations. Each mutation is represented by a bubble whose size is scaled on the *alt_depth*. Thus, a large multiplet (i.e. high *alt_depth*) has a large bubble. This plot makes it easy to determine if MF_Max_ is largely driven by a few large multiplets or several moderate multiplets. Within the control, we observed a slight difference between MF_Min_ and MF_Max_, driven by three relatively large multiplets: two C: G > T: A mutations, and one deletion. In contrast, within the BaP dose groups, we observed that multiplets seemed to be evenly distributed, with no single mutation driving up the MF_Max_.

## 4 Discussion

Herein, we demonstrate the utility of MutSeqR for analyzing ECS data in the assessment of chemical mutagenicity. MutSeqR successfully recapitulates published findings (LeBlanc *et al.* 2022), replacing previously used *ad hoc* scripts with a version-controlled, function-based workflow. Furthermore, we demonstrate that MutSeqR readily imports data from distinct platforms, illustrating this feature with DS and SMM-seq data.

MutSeqR provides a comprehensive, user-friendly, and reproducible framework for analyzing ECS data in mutagenicity studies, making it a valuable tool to the regulatory community. It offers practical variant filtering methods to ensure data quality, providing a strong foundation for downstream analyses. We provide functionality for statistical testing between experimental groups and BMD derivation for a given mutagen. In a regulatory use case, these features deliver quantitative data on a substance’s mutagenic ability, directly informing risk assessment decisions. The package also supports mutation spectrum analysis and signature assignment, offering insight into a mutagen’s mechanistic action and potentially linking mutagen exposure with established human health risks like cancer. Furthermore, data visualization facilitates clear communication and transparent reporting, allowing regulators to interpret complex findings efficiently and justify their decisions to stakeholders with confidence. Finally, to enhance reproducibility, an RMarkdown vignette guides users through standard workflows and serves as a template for consistent and verifiable data analysis. We used preliminary, less formalized versions of this code in our previous ECS studies (LeBlanc *et al.* 2022, Dodge *et al.* 2023, Cho *et al.* 2023, Esina *et al.* 2024, Schuster *et al.* 2024). MutSeqR formalizes these workflows, making them accessible, version-controlled, and easily adapted to any ECS project, thereby strengthening the workflow for regulatory genotoxicity evaluations.

Outside of the use cases considered here, it is important to recognize that MutSeqR’s utility can extend beyond mutagenicity assessment. ECS methods are rapidly being developed to study a broad range of genetic toxicology endpoints, not just MF and spectra. For instance, applications in carcinogenesis have begun to rely on ECS to track the clonal expansion of cells carrying mutated cancer driver genes. MutSeqR provides users with the ability to analyse clonal mutations using the Maximum Mutation count and the ability to visualize such mutations using *plot_bubbles()*.

Given the increased use of ECS in genetic toxicology (Valentine *et al.* 2020, LeBlanc *et al.* 2022, Dodge *et al.* 2023, Marchetti *et al.* 2023, Smith-Roe *et al.* 2023, Schuster *et al.* 2024), the statistical techniques captured within and simplified by MutSeqR provide a valuable resource for efficient and reproducible analysis of mutation data. Thanks to its compatibility across ECS platforms, MutSeqR will help standardize the bioinformatic workflow involved with characterizing mutations using ECS and act as a guide to help unite the protocols for data processing of the many different ECS strategies. Ultimately, our vision is that this package informs a standardized approach to ECS data processing and analysis that can be applied within a regulatory and research context. Future development will focus on integrating the fastquorum pipeline for upstream variant calling (based on fgbio; https://nf-co.re/fastquorum/) and expanding the available mutational signatures by incorporating databases such as SIGNAL. We also plan to implement additional exploratory analyses, such as transcriptional strand bias tests and screening for extra-chromosomal circular DNA. MutSeqR will remain openly available on GitHub and will continue to evolve through transparent collaboration between maintainers and the broader community to address future challenges in ECS data analysis.

## Supplementary Material

vbaf265_Supplementary_Data

## Data Availability

The data underlying this article are available in *GitHub* at https://github.com/EHSRB-BSRSE-Bioinformatics/MutSeqR.

## References

[vbaf265-B1] Abbasi A , Alexandrov LB. Significance and limitations of the use of next-generation sequencing technologies for detecting mutational signatures. DNA Repair (Amst) 2021;107:103200. 10.1016/j.dnarep.2021.103200 34411908 PMC9478565

[vbaf265-B2] Adesina OA , Olowolafe TI, Igbafe A. Levels of polycyclic aromatic hydrocarbon from mainstream smoke of tobacco products and its risks assessment. J Hazard Mater Adv 2022;5:100053. 10.1016/j.hazadv.2022.100053

[vbaf265-B3] Beal MA , Gagné R, Williams A et al Characterizing benzo[a]pyrene-induced lacZ mutation spectrum in transgenic mice using next-generation sequencing. BMC Genomics 2015;16:812. 10.1186/s12864-015-2004-4 26481219 PMC4617527

[vbaf265-B4] Beal MA , Yauk CL, Marchetti F. From sperm to offspring: assessing the heritable genetic consequences of paternal smoking and potential public health impacts. Mutat Res Rev Mutat Res 2017;773:26–50. 10.1016/j.mrrev.2017.04.001 28927533

[vbaf265-B5] Bercu JP , Zhang S, Sobol Z et al Comparison of the transgenic rodent mutation assay, error corrected next generation duplex sequencing, and the alkaline comet assay to detect dose-related mutations following exposure to N-nitrosodiethylamine. Mutat Res Genet Toxicol Environ Mutagen 2023;891:503685. 10.1016/j.mrgentox.2023.503685 37770142

[vbaf265-B7] Cho E , Swartz CD, Williams A et al Error-corrected duplex sequencing enables direct detection and quantification of mutations in human TK6 cells with strong inter-laboratory consistency. Mutat Res Genet Toxicol Environ Mutagen 2023;889:503649. 10.1016/j.mrgentox.2023.503649 37491114 PMC10395007

[vbaf265-B8] Danecek P , Auton A, Abecasis G et al; 1000 Genomes Project Analysis Group. The variant call format and VCFtools. Bioinformatics 2011;27:2156–8. 10.1093/bioinformatics/btr330 21653522 PMC3137218

[vbaf265-B9] Díaz-Gay M , Vangara R, Barnes M et al Assigning mutational signatures to individual samples and individual somatic mutations with SigProfilerAssignment. Bioinformatics 2023;39:btad756. 10.1093/bioinformatics/btad756 38096571 PMC10746860

[vbaf265-B10] Dodge AE , LeBlanc DPM, Zhou G et al Duplex sequencing provides detailed characterization of mutation frequencies and spectra in the bone marrow of MutaMouse males exposed to procarbazine hydrochloride. Arch Toxicol 2023;97:2245–59. 10.1007/s00204-023-03527-y 37341741 PMC10322784

[vbaf265-B11] Erickson RP. Somatic gene mutation and human disease other than cancer. Mutat Res 2003;543:125–36. 10.1016/s1383-5742(03)00010-3 12644182

[vbaf265-B12] Esina E , Dodge AE, Williams A et al Power analyses to inform duplex sequencing study designs for MutaMouse liver and bone marrow. Environ Mol Mutagen 2024;65:234–42. 10.1002/em.22619 39267335

[vbaf265-B13] Everitt B. Cluster Analysis. London: Heinemann Educational Books Ltd., 1974.

[vbaf265-B14] Fung KY , Douglas GR, Krewski D. Statistical analysis of lacZ mutant frequency data from MutaMouse mutagenicity assays. Mutagenesis 1998;13:249–55. 10.1093/mutage/13.3.249 9643583

[vbaf265-B16] Godschalk RWL , Yauk CL, van Benthem J et al In utero exposure to genotoxicants leading to genetic mosaicism: an overlooked window of susceptibility in genetic toxicology testing? Environ Mol Mutagen 2020;61:55–65. 10.1002/em.22347 31743493 PMC6973016

[vbaf265-B17] Halekoh U , Højsgaard S. doBy: Groupwise Statistics, LSmeans, Linear Estimates, Utilities. Version 4.6.25 [R package]. https://CRAN.R-project.org/package=doBy. 2025.

[vbaf265-B18] Hartigan JA. Clustering Algorithms. Hoboken: John Wiley & Sons, 1975.

[vbaf265-B19] Jackson M , Marks L, May GHW et al The genetic basis of disease. Essays Biochem 2018;62:643–723. 10.1042/EBC20170053 30509934 PMC6279436

[vbaf265-B100] Kennedy SR , Schmitt MW, Fox EJ et al Detecting ultralow-frequency mutations by Duplex Sequencing. Nat Protoc 2014;9:2586–606. 10.1038/nprot.2014.170 25299156 PMC4271547

[vbaf265-B20] Kennedy SR , Zhang Y, Risques RA. Cancer-associated mutations but no cancer: insights into the early steps of carcinogenesis and implications for early cancer detection. Trends Cancer 2019;5:531–40. 10.1016/j.trecan.2019.07.007 31474358 PMC8765002

[vbaf265-B21] Khandekar A , Vangara R, Barnes M et al Visualizing and exploring patterns of large mutational events with SigProfilerMatrixGenerator. BMC Genomics 2023;24:469. 10.1186/s12864-023-09584-y 37605126 PMC10440861

[vbaf265-B22] Kostecka A , Nowikiewicz T, Olszewski P et al High prevalence of somatic PIK3CA and TP53 pathogenic variants in the normal mammary gland tissue of sporadic breast cancer patients revealed by duplex sequencing. NPJ Breast Cancer 2022;8:76–10. 10.1038/s41523-022-00443-9 35768433 PMC9243094

[vbaf265-B23] Krewski D , Acosta D, Andersen M et al Toxicity testing in the 21st century: a vision and a strategy. J Toxicol Environ Health B Crit Rev 2010;13:51–138. 10.1080/10937404.2010.483176 20574894 PMC4410863

[vbaf265-B24] Kucab JE , Zou X, Morganella S et al A compendium of mutational signatures of environmental agents. Cell 2019;177:821–36.e16. 10.1016/j.cell.2019.03.001 30982602 PMC6506336

[vbaf265-B25] Lai Z , Markovets A, Ahdesmaki M et al VarDict: a novel and versatile variant caller for next-generation sequencing in cancer research. Nucleic Acids Res 2016;44:e108. 10.1093/nar/gkw227 27060149 PMC4914105

[vbaf265-B26] Lawrence M , Huber W, Pagès H et al Software for computing and annotating genomic ranges. PLoS Comput Biol 2013;9:e1003118. 10.1371/journal.pcbi.1003118 23950696 PMC3738458

[vbaf265-B27] LeBlanc DPM , Meier M, Lo FY et al Duplex sequencing identifies genomic features that determine susceptibility to benzo(a)pyrene-induced in vivo mutations. BMC Genomics 2022;23:542. 10.1186/s12864-022-08752-w 35902794 PMC9331077

[vbaf265-B28] Liamin M , Boutet-Robinet E, Jamin EL et al Benzo[a]pyrene-induced DNA damage associated with mutagenesis in primary human activated T lymphocytes. Biochem Pharmacol 2017;137:113–24. 10.1016/j.bcp.2017.04.025 28461126

[vbaf265-B29] Marchetti F , Douglas GR, Yauk CL. A return to the origin of the EMGS: rejuvenating the quest for human germ cell mutagens and determining the risk to future generations. Environ Mol Mutagen 2020;61:42–54. 10.1002/em.22327 31472026

[vbaf265-B30] Marchetti F , Cardoso R, Chen CL et al Error-corrected next generation sequencing—promises and challenges for genotoxicity and cancer risk assessment. Mutat Res Rev Mutat Res 2023;792:108466. 10.1016/j.mrrev.2023.108466 37643677

[vbaf265-B31] Maslov AY , Makhortov S, Sun S et al Single-molecule, quantitative detection of low-abundance somatic mutations by high-throughput sequencing. Sci Adv 2022;8:eabm3259. 10.1126/sciadv.abm3259 35394831 PMC8993124

[vbaf265-B32] Menon V , Brash DE. Next-generation sequencing methodologies to detect low-frequency mutations: “catch me if you can. Mutat Res Rev Mutat Res 2023;792:108471. 10.1016/j.mrrev.2023.108471 37716438 PMC10843083

[vbaf265-B33] More SJ , Bampidis V, Benford D et al; EFSA Scientific Committee. Guidance on the use of the benchmark dose approach in risk assessment. EFSA J 2022;20:e07584. 10.2903/j.efsa.2022.7584 36304832 PMC9593753

[vbaf265-B34] Nguengang Wakap S , Lambert DM, Olry A et al Estimating cumulative point prevalence of rare diseases: analysis of the orphanet database. Eur J Hum Genet 2020;28:165–73. 10.1038/s41431-019-0508-0 31527858 PMC6974615

[vbaf265-B35] Piegorsch WW , Bailer AJ. Statistical approaches for analyzing mutational spectra: some recommendations for categorical data. Genetics 1994;136:403–16. 10.1093/genetics/136.1.403 8138174 PMC1205789

[vbaf265-B36] Salk JJ , Kennedy SR. Next‐generation genotoxicology: using modern sequencing technologies to assess somatic mutagenesis and cancer risk. Environ Mol Mutagen 2020;61:135–51. 10.1002/em.22342 31595553 PMC7003768

[vbaf265-B37] Schuster DM , LeBlanc DPM, Zhou G, et al Dose-related mutagenic and clastogenic effects of benzo[b]fluoranthene in mouse somatic tissues detected by duplex sequencing and the micronucleus assay. Environ Sci Technol 2024;58:21450–63. 10.1101/2024.07.26.605228 39602390 PMC11636207

[vbaf265-B38] Seplyarskiy VB , Soldatov RA, Koch E et al; TOPMed Population Genetics Working Group. Population sequencing data reveal a compendium of mutational processes in the human germ line. Science (1979) 2021;373:1030–5. 10.1126/science.aba7408 PMC9217108 34385354

[vbaf265-B39] Smith-Roe SL , Hobbs CA, Hull V et al Adopting Duplex Sequencing^TM^ technology for genetic toxicity testing: a proof-of-concept mutagenesis experiment with N-ethyl-N-nitrosourea (ENU)-exposed rats. bioRxiv, 10.1101/2023.05.08.539833, 2023, preprint: not peer reviewed. PMC10539650 37770135

[vbaf265-B40] Valentine CC , Young RR, Fielden MR et al Direct quantification of in vivo mutagenesis and carcinogenesis using duplex sequencing. Proc Natl Acad Sci USA 2020;117:33414–25. 10.1073/pnas.2013724117 33318186 PMC7776782

[vbaf265-B44] White PA , Long AS, Johnson GE. Quantitative interpretation of genetic toxicity dose‐response data for risk assessment and regulatory decision‐making: current status and emerging priorities. Environ Mol Mutagen 2020;61:66–83. 10.1002/em.22351 31794061

[vbaf265-B45] Zou X , Koh GCC, Nanda AS et al; Genomics England Research Consortium. A systematic CRISPR screen defines mutational mechanisms underpinning signatures caused by replication errors and endogenous DNA damage. Nat Cancer 2021;2:643–57. 10.1038/s43018-021-00200-0 34164627 PMC7611045

