# Supplementary material for: MutSeqR: an open source R package for standardized analysis of error-corrected next-generation sequencing data in genetic toxicology
Source: Bioinform Adv. 2025 Oct 23;5(1):vbaf265. doi: 10.1093/bioadv/vbaf265 (PMC12645840; doi:10.1093/bioadv/vbaf265)
Supplement: vbaf265_Supplementary_Data [file vbaf265_supplementary_data.zip › MutSeqR Supplementary Material 20251001_revised - Clean.docx]

Supplementary Material

Supplementary Tables

**Supplementary Table 1**. Definitions of variation type categories.

| Variation type |  | Definition |
| --- | --- | --- |
| no_variant |  | No variation; the null case |
| snv | Single Nucleotide Variant | DNA sequence variation that occurs when a single nucleotide in the genome sequence is substituted for another nucleotide. |
| mnv | Multi-Nucleotide Variant | DNA sequence variation that occurs when two or more consecutive nucleotides in the genome sequence are substituted for other nucleotides. |
| insertion |  | DNA sequence variation that occurs when there is an addition of one or more nucleotides into a DNA sequence. |
| deletion |  | DNA sequence variation that occurs when there is a deletion of one or more nucleotides from a DNA sequence. |
| complex |  | DNA sequence variation that occurs when one or more nucleotides in the genome sequence are substituted for another nucleotide and there is also an addition or deletion of one or more nucleotides from the sequence. |
| sv | Structural Variant | A change in the DNA sequence that involves at least 50 bp. May include copy number variants (CNV), deletions (DEL), duplications (DUP), insertions (INS), inversions (INV), and translocations (TRA). |
| ambiguous | IUPAC ambiguity codes | A variation category that is called when one or more IUPAC ambiguity codes for nucleotide degeneracy are called within the alt column. Represents uncertainty in the sequencing data. |
| uncategorized |  | A variation category that is called when a given record does not fall into any of the above categories. |

**Supplementary Table 2**. Benchmark dose modelling of BaP on MF_Min_ by PROAST.

| Model | BMD | BMDL | BMDU | AIC | Weights |
| --- | --- | --- | --- | --- | --- |
| Exponential m5 | 8.45 | 6.47 | 10.3 | -18.46 | 0.3316 |
| Hill m5 | 9.03 | 7.02 | 10.7 | -18.46 | 0.3316 |
| Inverse Exponential m3 | 4.71 | 2.9 | 6.96 | -10.18 | 0.0053 |
| Lognormal m5 | 9.17 | 7.27 | 10.7 | -18.46 | 0.3316 |
| Model Averaging | 9.11 | 7.38 | 10.9 |  |  |

**Supplementary Table 3.** Benchmark dose modelling of BbF on MF_Min_ by PROAST.

| Model | BMD | BMDL | BMDU | AIC | Weights |
| --- | --- | --- | --- | --- | --- |
| Exponential m5 | 31.12 | 20.9 | 41.9 | -2.5 | 0.297 |
| Hill m5 | 30.34 | 20.2 | 41.2 | -2.2 | 0.2556 |
| Inverse Exponential m3 | 27.47 | 18.4 | 40 | -1.82 | 0.2114 |
| Lognormal m5 | 28.8 | 19.3 | 40.8 | -2.04 | 0.236 |
| Model Averaging | 28.8 | 17.0 | 39.4 |  |  |

Supplementary Figures


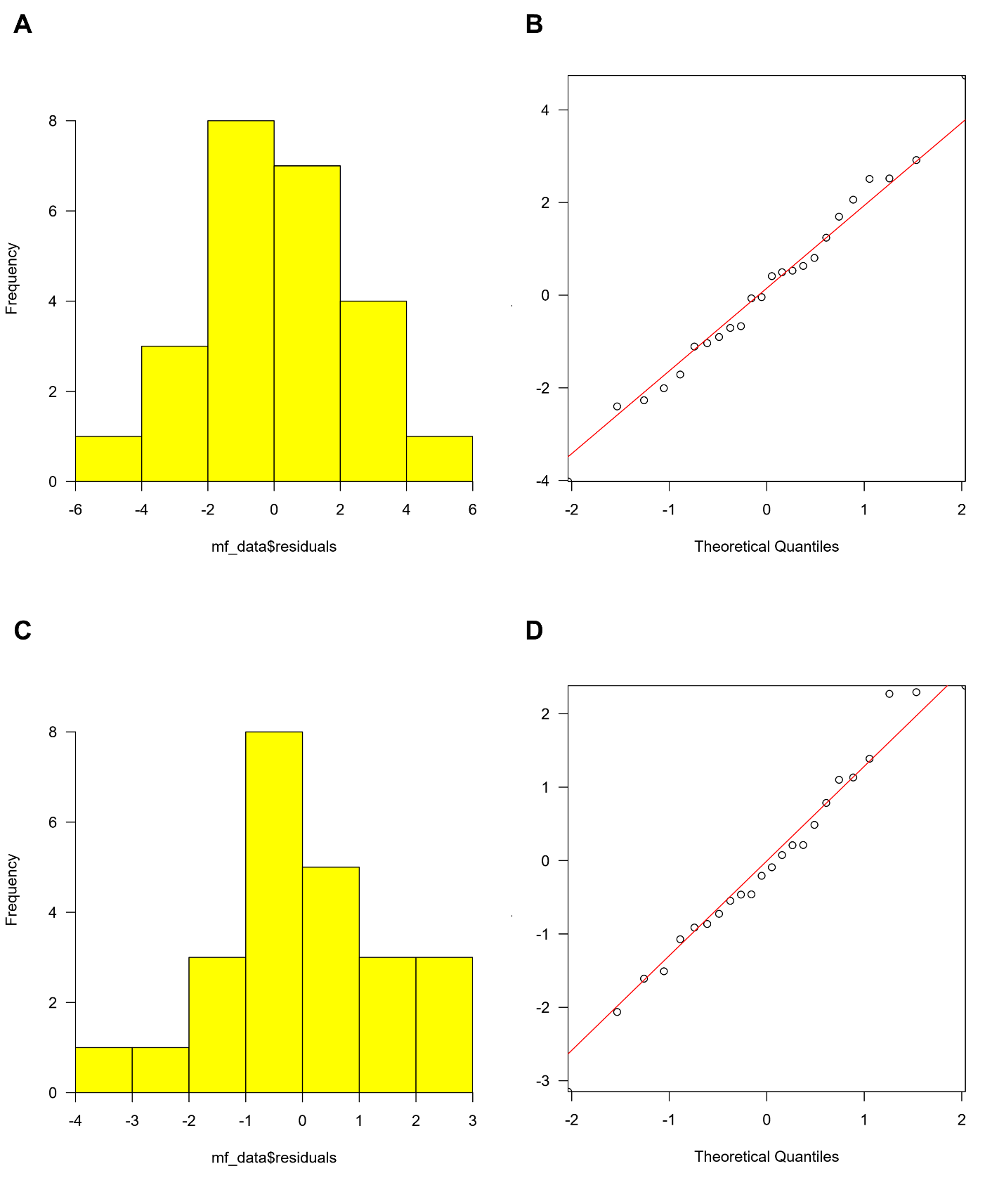


**Supplementary Figure 1.** Model residuals of MF_Min_ modelled as an effect of chemical dose, plotted to check model assumptions. A) Project 1: Histogram of residuals for the effect of BaP on MF_Min_, n = 24. A histogram has model residuals on the x-axis and their frequency on the y-axis. Normally distributed residuals form a bell curve. B) Project 1: Quantile-Quantile (QQ) plot of residuals for the effect of BaP on MF_Min_. QQ plots plot the model residuals in ascending order on the y-axis (Sample Quantiles) and the n = n(samples) quantiles of the standard normal distribution on the x-axis (Theoretical Quantiles). Normally distributed residuals are plotted as a straight line. C) Project 2: Histogram of residuals for effect of BbF on MF_Min_ n = 24. D) Project 2: QQ plot of residuals for effect of BbF on MF_Min_. Plotted using *model_mf()*.


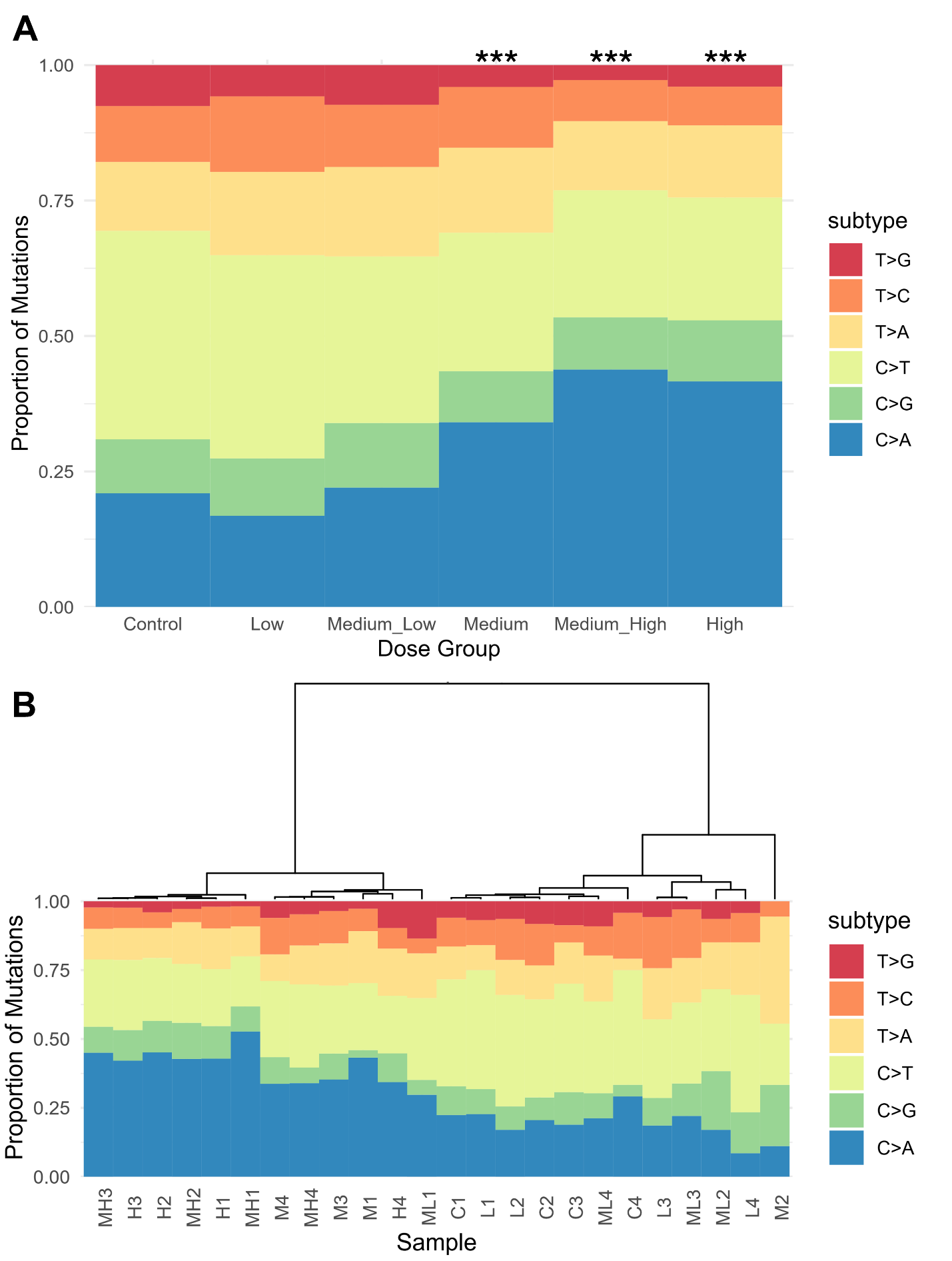


**Supplementary Figure 2.** A) Mutation spectrum of control and BbF dose groups measured by SMM-Seq in the liver of MutaMouse males. Proportions of mutation subtypes are represented by colour within the stacked bar for each BbF dose group. Asterisks indicate a significant difference in the mutation spectra compared to the control (*spectra_comparison()*; modified contingency table, p<0.05). Plotted using *plot_spectra()*, asterisks added separately. B) Mutation spectrum of individual animals measured by SMM-Seq. Animals are clustered into groups based on the Euclidean distance between their subtype proportions using *cluster_spectra()*. Sample identifiers denote dose group: C – Control, L – Low, ML – Medium low, M – Medium, MH – Medium high, H – High. Plotted with *plot_spectra.*

*
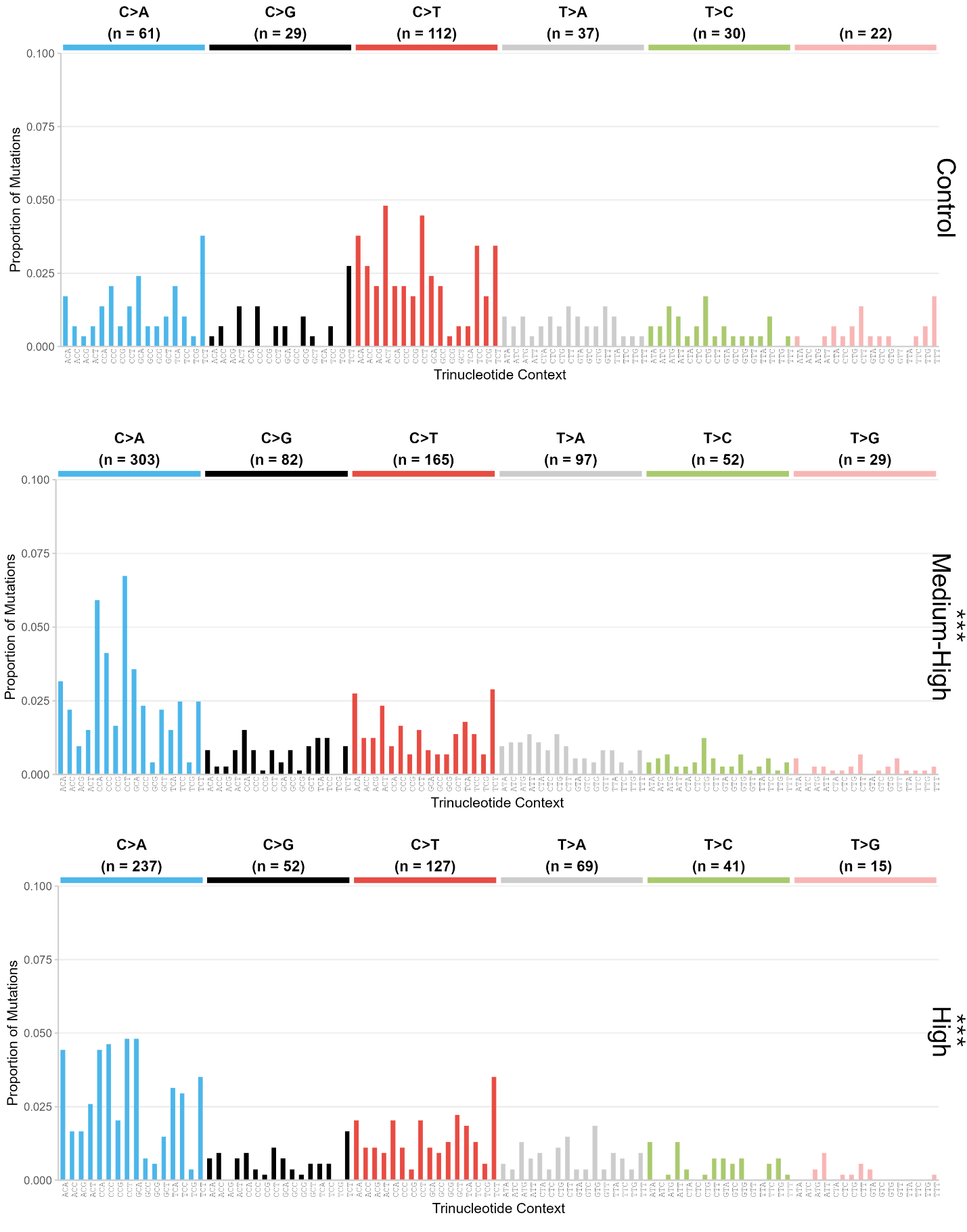
*

**Supplementary Figure 3.** Proportion of mutation subtypes within their 96-trinucleotide context for control, medium-high, and high dose BbF dose groups, measured by SMM-Seq. Bars are coloured based on the normalized SNV subtype. Data labels represent the number of mutations for each normalized SNV subtype within that dose group. Asterisks indicate significant differences in spectra from control (p < 0.05). Plotted using *plot_trinucleotide()*; dose group labels and asterisks were added separately.


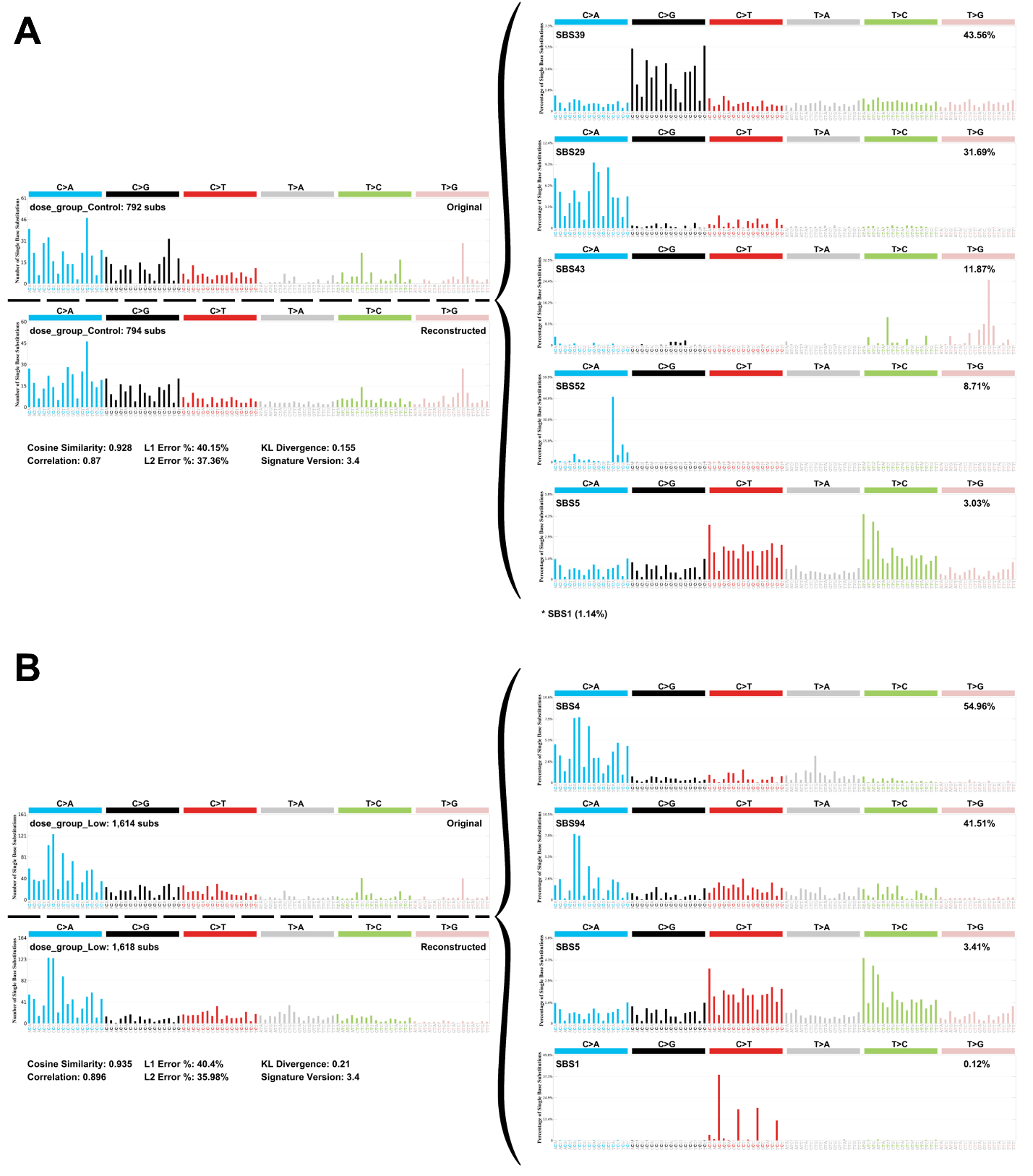


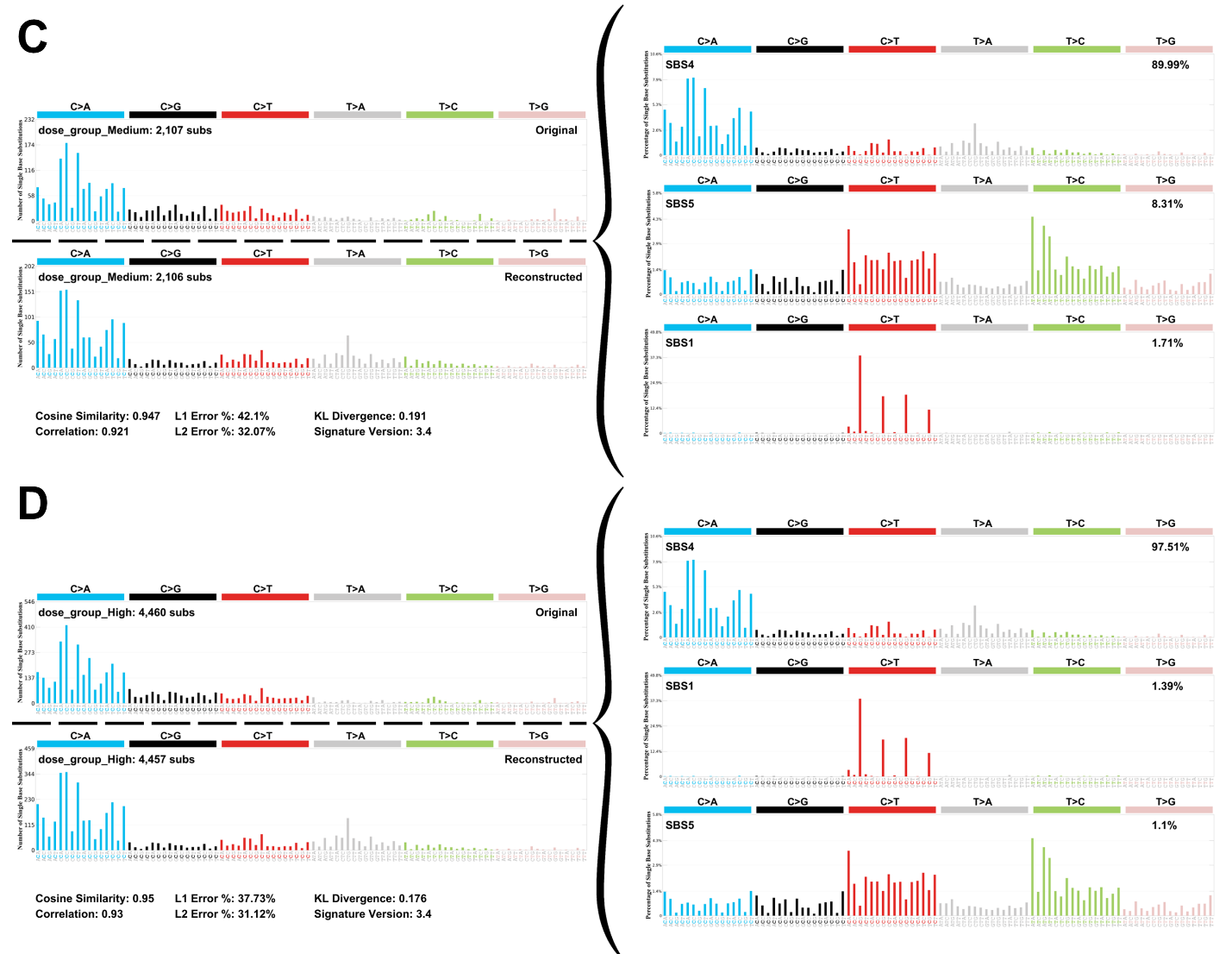


**Supplementary Figure 4.** Mutation signature analysis for A) control, B) Low, C) Medium, and D) High BaP dose groups in the bone marrow of MutaMouse males. The original trinucleotide mutation profile is shown on the top left. *SigProfilerAssignment* used the single base substitution (SBS) signatures of the Catalogue of Somatic Mutations in Cancer (COSMIC) database to reconstruct the mutational profile (bottom left). The SBS signatures and their relative contributions are shown on the right. Solution statistics for the reconstruction are displayed on the bottom left, including the cosine similarity between the reconstructed and observed mutation profile. The total number of mutations in the original mutation profile is indicated on the top left of the profile plot. Plotted using *SigProfilerAssignment* using a data structure, virtual environment created by MutSeqR, and MutSeqR’s *signature_fitting()* function.


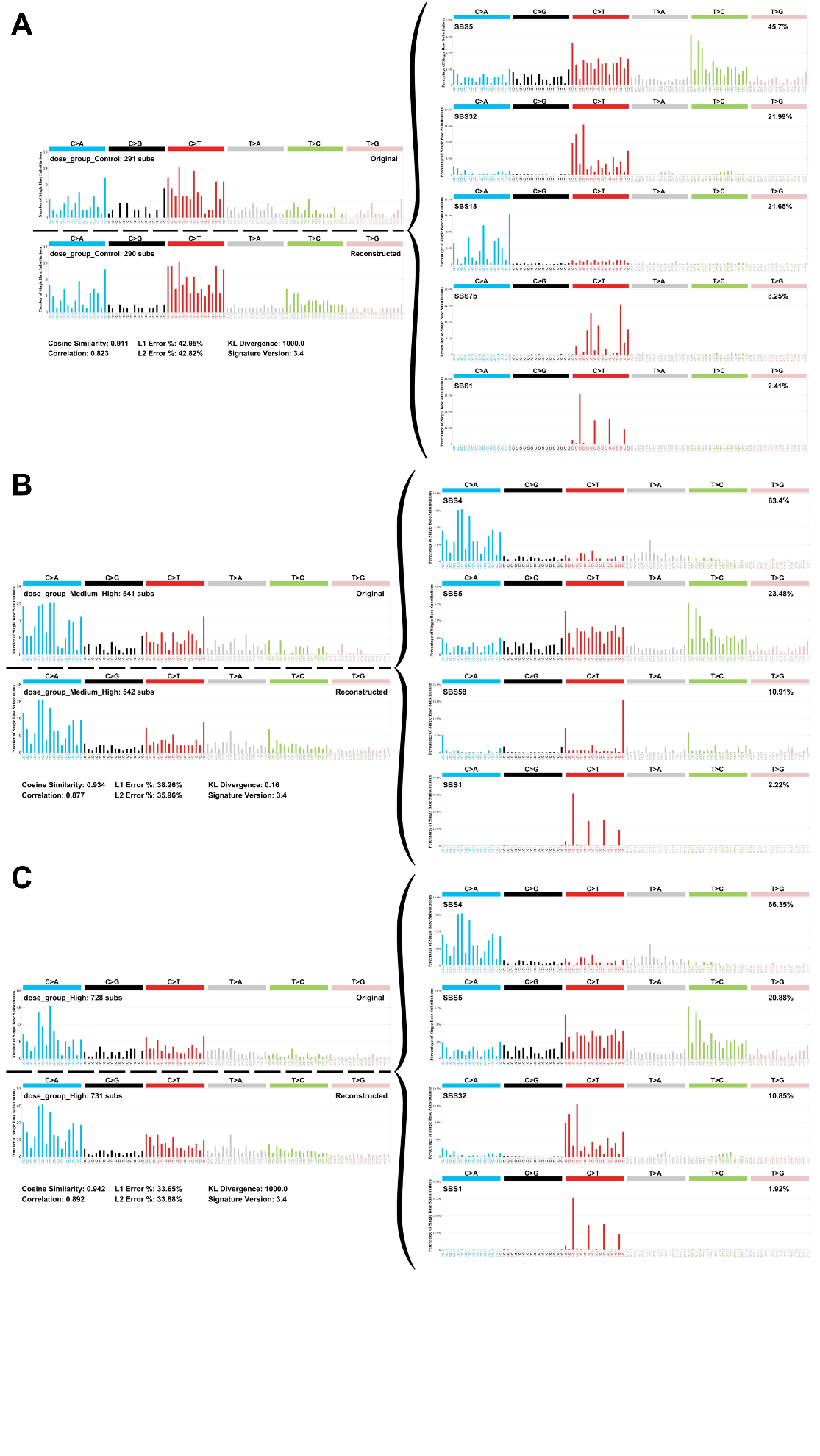


**Supplementary Figure 5.** Mutation signature analysis for A) control, B) medium-high and C) high BbF dose groups in the liver of MutaMouse males. The original trinucleotide mutation profile is shown on the top left. *SigProfilerAssignment* used the single base substitution (SBS) signatures of the Catalogue of Somatic Mutations in Cancer (COSMIC) database to reconstruct the mutational profile (bottom left). The SBS signatures and their relative contributions are shown on the right. Solution statistics for the reconstruction are displayed on the bottom left, including the cosine similarity between the reconstructed and observed mutation profile. The total number of mutations in the original mutation profile is indicated on the top left of the profile plot. Plotted using *SigProfilerAssignment* using a data structure, virtual environment created by MutSeqR, and MutSeqR’s *signature_fitting()* function.

**
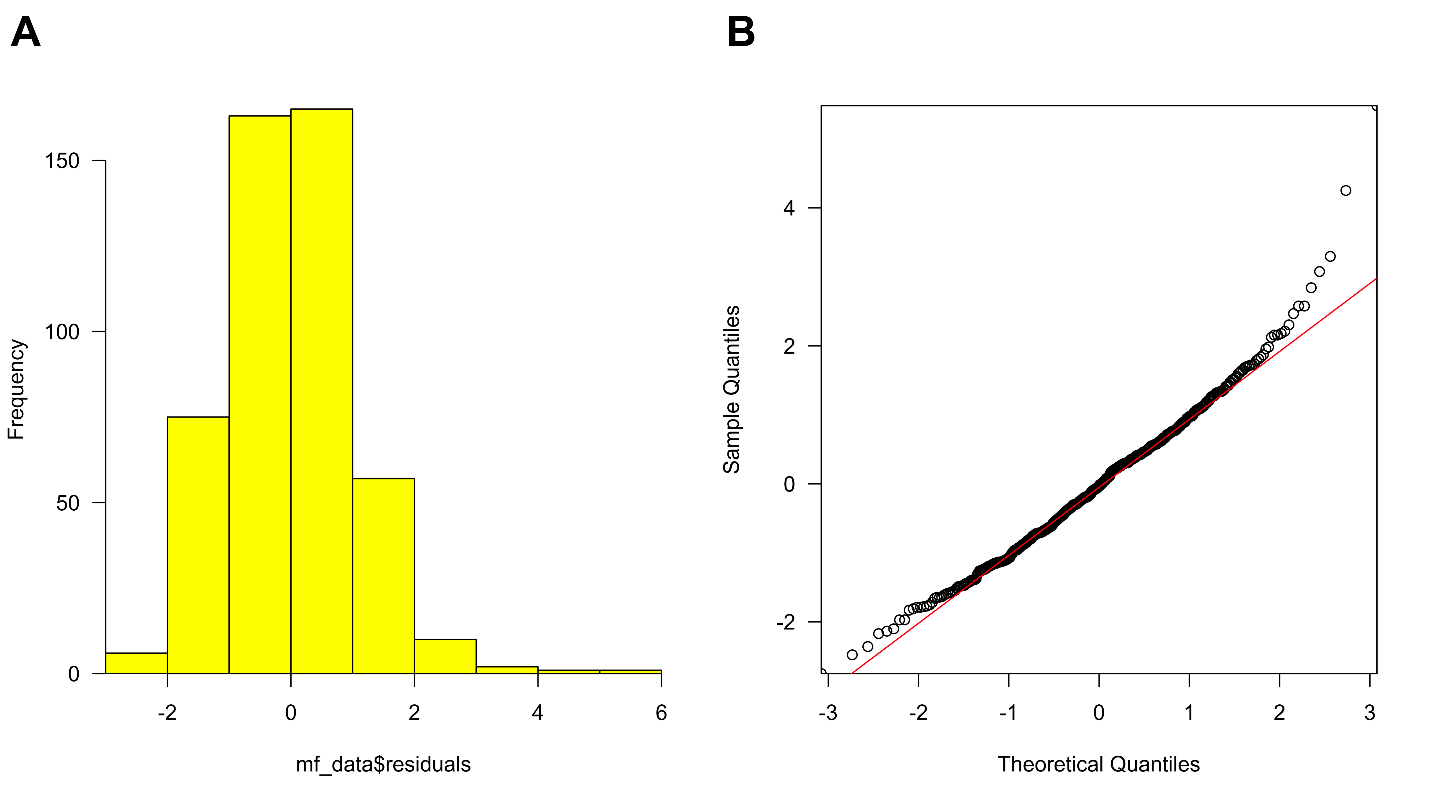
Supplementary Figure 6.** Model residuals plotted to check model assumptions. Residuals of MF_Min_ modelled as an effect of BaP dose and Duplex Sequencing target, n = 480 (24 samples * 20 targets). A) A histogram has model residuals on the x-axis and their frequency on the y-axis. Normally distributed residuals form a bell curve. B) Quantile-quantile (QQ) plots plot the model residuals in ascending order on the y-axis (Sample Quantiles) and the n = n(data points) quantiles of the standard normal distribution on the x-axis (Theoretical Quantiles). Normally distributed residuals are plotted as a straight line. Plotted using *model_mf()*.
